# The Genetic Structure of the Swedish Population

**DOI:** 10.1371/journal.pone.0022547

**Published:** 2011-08-04

**Authors:** Keith Humphreys, Alexander Grankvist, Monica Leu, Per Hall, Jianjun Liu, Samuli Ripatti, Karola Rehnström, Leif Groop, Lars Klareskog, Bo Ding, Henrik Grönberg, Jianfeng Xu, Nancy L. Pedersen, Paul Lichtenstein, Morten Mattingsdal, Ole A. Andreassen, Colm O'Dushlaine, Shaun M. Purcell, Pamela Sklar, Patrick F. Sullivan, Christina M. Hultman, Juni Palmgren, Patrik K. E. Magnusson

**Affiliations:** 1 Department of Medical Epidemiology and Biostatistics, Karolinska Institutet, Stockholm, Sweden; 2 Human Genetics Laboratory, Genome Institute of Singapore, Singapore, Singapore; 3 Institute for Molecular Medicine, Finland, FIMM, University of Helsinki, Helsinki, Finland; 4 Public Health Genomics Unit, National Institute for Health and Welfare, Helsinki, Finland; 5 Wellcome Trust Sanger Institute, Wellcome Trust Genome Campus, Hinxton, United Kingdom; 6 Department of Clinical Sciences, Diabetes and Endocrinology, Lund University Diabetes Centre, Malmö, Sweden; 7 Institute of Environmental Medicine, Karolinska Institutet, Stockholm, Sweden; 8 Department of Cancer Biology and Comprehensive Cancer Center, Wake Forest University School of Medicine, Winston-Salem, North Carolina, United States of America; 9 Center for Cancer Genomics, Wake Forest University School of Medicine, Winston-Salem, North Carolina, United States of America; 10 Institute of Clinical Medicine, Section Psychiatry, University of Oslo, Oslo, Norway; 11 Sørlandet Hospital HF, Kristiansand, Norway; 12 Division of Mental Health and Addiction, Oslo University Hospital, Oslo, Norway; 13 Broad Institute of MIT and Harvard, Cambridge, Massachusetts, United States of America; 14 Psychiatric and Neurodevelopmental Genetics Unit, Center for Human Genetic Research, Massachusetts General Hospital, Boston, Massachusetts, United States of America; 15 Department of Genetics, University of North Carolina at Chapel Hill, Chapel Hill, North Carolina, United States of America; 16 Department of Mathematical Statistics, Stockholm University, Stockholm, Sweden; 17 Swedish eScience Research Center, Stockholm, Sweden; The Centre for Research and Technology, Greece

## Abstract

Patterns of genetic diversity have previously been shown to mirror geography on a global scale and within continents and individual countries. Using genome-wide SNP data on 5174 Swedes with extensive geographical coverage, we analyzed the genetic structure of the Swedish population. We observed strong differences between the far northern counties and the remaining counties. The population of *Dalarna county*, in north middle Sweden, which borders southern Norway, also appears to differ markedly from other counties, possibly due to this county having more individuals with remote Finnish or Norwegian ancestry than other counties. An analysis of genetic differentiation (based on pairwise F_st_) indicated that the population of Sweden's southernmost counties are genetically closer to the HapMap CEU samples of Northern European ancestry than to the populations of Sweden's northernmost counties. In a comparison of extended homozygous segments, we detected a clear divide between southern and northern Sweden with small differences between the southern counties and considerably more segments in northern Sweden. Both the increased degree of homozygosity in the north and the large genetic differences between the south and the north may have arisen due to a small population in the north and the vast geographical distances between towns and villages in the north, in contrast to the more densely settled southern parts of Sweden. Our findings have implications for future genome-wide association studies (GWAS) with respect to the matching of cases and controls and the need for within-county matching. We have shown that genetic differences within a single country may be substantial, even when viewed on a European scale. Thus, population stratification needs to be accounted for, even within a country like Sweden, which is often perceived to be relatively homogenous and a favourable resource for genetic mapping, otherwise inferences based on genetic data may lead to false conclusions.

## Introduction

It is important to understand the genetic gradients and stratifications within countries, from both population genetic and medical perspectives. Genetic gradients are represented by differences in allele frequencies, which have come about due to events such as migration, genetic drift or differential selection [1]. On a European scale, the gradients have been shown to correspond well to geography [2], [3], [4], [5], [6] and may be useful for understanding population structure on a fine-spatial scale. Previous studies have shown considerable genetic substructure within Finland [7] that has arisen due to multiple isolated populations with late settlement and a small founder population. To a lesser extent, intra-country genetic differences have also been shown within the UK [8], [9], Switzerland [4], Estonia and Italy [10], and Iceland [11], although with considerable overlap between sub-populations and with less distinct differences than between eastern and western Finland.

The Swedish population has been reported to be genetically similar to the nearby populations of Norway, Denmark and Germany, with which there have been long term interaction, but markedly less similar to the Finnish population, although Finland is geographically close [4], [5], [10]. Finland and Sweden have a long common history and were one country until 1809, geographically separated to a large extent by the Baltic Sea but bordered by land in the far north. Finns make up the largest ethnic minority in Sweden (about 5% of the Swedish population), having come both before 1809 and in large numbers during the 20^th^ century. Population densities, which impact on population structure, vary greatly across Sweden (Table S1).

Previous studies have shown weak population structure among Swedes, i.e. clinal trends of variation [12]. These studies have typically been based on data with much lower resolution than what the highly multiplexed genotyping chips currently used to assay genome-wide variation offer. For example, Hannelius et al. [12] genotyped 34 unlinked autosomal single nucleotide polymorphisms, originally designed for zygosity testing, from 2044 samples from Sweden. One recent study [13] was based on 350,000 SNPs from 1525 Swedes genotyped on the Illumina HumanHap550 arrays, with information on place of (current) residence, and described more pronounced genetic differences within Sweden, underlining the potential of genomewide data for analysing substructure in detail. In the present study we have access to a much larger number of samples, of which many have information on residence at birth.

In population-based genetic association studies it is important to have a genetically homogenous population from which to sample cases and controls, both for detecting true associations, to avoid reductions in power due to genetic stratification between the case and control groups, and to avoid false-positive findings. Cases and controls are almost always matched with respect to the country-of-origin, but a finer-scale matching on a sub-country basis may be necessary. In most of the GWAS performed to date, genetic stratification between the case and control groups has been present, as indicated by the genomic control lambda being above 1.0 (i.e. a median p-value below 0.5), e.g. the first Wellcome Trust Case-Control Study [8]. The understanding of population genetic differences could help researchers in their selection of study participants in order to get better matching between groups and to control for known genetic gradients when perfect matching is not achieved.

Swedes have been included in a wide variety of GWAS, e.g. type 2 diabetes, breast cancer, prostate cancer, schizophrenia, and bipolar disorder. Sweden has a population of only 9 million, a 40-year history of nation-wide medical registers, and high research study participation rates in an almost exclusively publicly funded healthcare system. This has made the collection of substantial numbers of cases possible, even for rare diseases.

We have investigated the Swedish genetic structure and its effect on population-based genetic studies in Sweden using a comprehensive collection of more than 5000 control samples collected across the country. We have used information on each subject's village or city of birth, county of birth or county of current residence to link genetic structure to geography.

## Methods

### Data sources

Our data consist of controls (healthy individuals) collected in Sweden for GWAS on breast cancer (CAHRES; CAncer Hormone Replacement Epidemiology in Sweden), prostate cancer (CAPS; Cancer Prostate in Sweden), diabetes type 2 (DGI; Diabetes Genetics Initiative), schizophrenia (CSZ-SW; A Swedish Population based Case-Control genetic study in Schizophrenia), rheumatoid arthritis (EIRA; Epidemiological Investigation of Rheumatoid Arthritis) and an unselected twin-cohort study (TWINGENE-SW; Swedish Twin Registry Genetic studies of common diseases), that were typed on platforms from Illumina and Affymetrix (Table 1). Some of the datasets were retrieved from the Nordic GWAS control database NordicDB [14]. Since the majority of samples were typed on Affymetrix platforms, which have a large number of overlapping SNPs, we chose to use the raw data on those samples and to impute the data for samples typed on Illumina platforms. Imputations were based on the program *Impute* version 1 [15] using the HapMap 2 CEU founders release 22 as reference [16].

**Table 1:** Description of included control samples.

Study	N*_samples_	Primary disease for which cases were selected	Genotyping platform
CAHRES [30]	732	Breast cancer	Illumina HumanHap 550
CAPS [31]	850	Prostate cancer	Affymetrix 550K and 5.0
DGI [23]	398	Diabetes type 2	Affymetrix 550K
SCZ-SW [32]	2290	Schizophrenia	Affymetrix 5.0 and 6.0
TWINGENE-SW [33]	290	Twin cohort study	Illumina HumanHap 300
EIRA [34]	614	Rheumatoid arthritis	Illumina HumanHap300
**Total**	5174		

*Numbers refer to the post-QC number of samples from each study.

### Geographical information

The county of residence was known for controls from the CAHRES and EIRA studies, while the county of birth was known for controls in the TWINGENE-SW study. For controls from the CAPS study, the municipality of residence was known (as well as county). For DGI the city of sample collection was known. For SCZ-SW the birthplace (city or village) and birth county was known. The best estimate of the geographical origin of each sample was assigned according to the following order: city or village of birth, county of birth, municipality or city of residence and county of residence. Coordinates for the locations were retrieved from the geographical location database Geonames [17].

### Handling of data sets and statistical analyses

Analyses and management of genotype data were carried out using *Plink* version 1.07 [18]. Plotting, simulations and statistical analyses were done in *R* version 2.10 [19]. The R package *maptools* was used for the plotting of maps [20].

### Merging of data sets

We first restricted all data sets to the 760,053 SNPs available in the largest sample set (SCZ-SW) that passed QC and whose alleles matched between data sets. 775 SNPs for which alleles differed between data sets, mainly alleles from imputed data sets with only one allele present, were removed prior to merging.

We then conducted extensive quality control of the SNPs in order to remove genotyping platform artifacts and study sample collection differences. We removed SNPs that had a genotyping success rate of less than 95% in any of the included studies. We also removed SNPs that had a minor allele frequency <0.01 in all studies combined or that failed Hardy-Weinberg equilibrium (with p<10^−6^) in all studies combined. In addition, we removed SNPs that differed between studies in a 1-vs-rest comparison (in which samples from one study were compared against all other studies) with p<10^−6^ in any comparison, in order to remove undetected strand flips (i.e., A-T and C-G flips) and SNPs differing due to technical differences between the studies. After QC, there were 184,449 autosomal SNPs for analysis.

### Initial sample QC

We removed samples that were too closely related to another sample (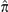
>0.20) and samples with genotype missingness>2%.

### Linkage disequilibrium pruning

The SNPs were pruned to be in approximate linkage equilibrium for the principal component analysis, the detection of extended homozygous segments and for the estimation of genetic differences between sub-populations. Plink was used for LD pruning, using a 200 SNP sliding window, a window step size of 25 SNPs and a maximum R^2^ threshold of 0.2 that was applied twice to remove SNPs in extended regions of high LD.

### Removal of samples with non-Swedish ancestry, principal component analysis and admixture analysis

All sample collection was carried out in Sweden but Swedes of foreign ancestry were eligible for inclusion in some of the studies. In order to avoid these samples confounding the analysis we sought to remove them, as the studies both had different inclusion criteria with respect to foreign ancestry and were sampled in different geographical locations. One or a few samples would not be able to distort the analysis but a larger number of samples, with common non-Swedish ancestry, might do so. Through questionnaire self-reporting we knew that a large number of samples in the SCZ-SW study had grandparental Finnish ancestry. As Finns previously have been shown to differ strikingly from other European populations [5] we sought to exclude the samples with Finnish ancestry. It may be argued that Finnish ancestry may be responsible for a large portion of the genetic stratification detectable in a Swedish sample but we chose to exclude them as the studies we combined had different inclusion criteria with respect to foreign ancestry. As the locations of sample collection also varied between the studies, the proportion of samples with Finnish ancestry in a county would not necessarily reflect the underlying proportion of inhabitants with Finnish ancestry in that county. Also, it is likely that the magnitude of stratification due to Finnish ancestry would overshadow any other subtle population structure patterns within Sweden. Since we did not have information on ancestry for subjects in other data sets other than SCZ-SW we had to rely on the genetic data to make exclusions. The fact that we had ancestral information on some samples made it possible for us to interpret the observed genetic patterns. Finnish ancestry was strongly correlated with the two first principal components (see results) and we defined an arbitrary, but strict, empirical cutoff, based on the two first components that removed samples of suspected Finnish ancestry. The principal component analysis was done using version 8000 of *smartpca* in the *Eigenstoft* package [21].

Samples with other non-Swedish ancestry were identified as outliers with respect to our population of samples, using the command –neighbor in Plink. For each sample we found the closest other sample (with respect to genetic distance) and transformed these distances to a standard-normal distribution. All samples that had a closest other sample more than 4 standard deviations from the mean were removed. This was repeated for the 2^nd^ to 5^th^ closest neighboring sample, in total removing 21 of the remaining samples.

We performed an analysis of admixture using the program ADMIXTURE [22] in an attempt to detect the presence of distinct ancestral populations within *Dalarna county*. We used the ten-fold cross-validation procedure, implemented within the program, to identify the number of “ancestral” populations for which the latent class model has best predictive accuracy.

### Genetic structure and non-primary data sets

The CEU founders from release 23 of HapMap were used in the comparison with the Swedish national areas and counties in the analysis of genetic differentiation. Pairwise F_st_ values were calculated using *smartpca* from the *Eigensoft* package. Genomic inflation λ values (ratios of the median of the empirically observed distribution of the test statistic to the expected median) for a study consisting of 1000 samples with cases and controls coming from two different regions (i.e. a fully stratified data set) were estimated from the F_st_ values according to: E(λ_GC 1000_) = 1+(1000 * F_st_) [5].

In order to investigate the second principal component, two additional data sets were used, one consisting of 939 controls collected in Western Finland as part of the DGI diabetes type 2 study [23] and one Norwegian consisting of 388 controls from the regions of Oslo and Akershus collected for the study of psychiatric disease [24], [25].

### Genomic inflation and power to detect association

In order to assess the effect of population stratification on the power of case-control studies to detect SNP association, we carried out a simulation study. We generated SNP data for repeated samples of 500 cases and 500 controls, under a variety of assumptions concerning minor allele frequencies (MAF), odds ratios (OR) and genomic inflation (λ). We calculated power to detect association based on using a genomewide significance threshold of 5×10^−8^.

### Detection of extended regions of homozygosity

We used Plink to call homozygous segments and used a 20 SNP sliding window that maximally could contain 1 heterozygous SNP if it was to be called homozygous. A SNP was determined to be in a homozygous segment if at least 10% of the overlapping windows were called as homozygous. We then filtered segments to be at least 1 Mb long, have a minimum of 50 SNPs and have no more than 5% heterozygous SNPs.

### Ethics statement

The appropriate regional ethics committees (DGI study: Lund University and Vaasa Central Hospital, CAPS study: Karolinska Institutet and Umeå University, EIRA study: Karolinska Institutet, CAHRES study: Karolinska Institutet, TWINGENE-SW: Karolinska Institutet, SCZ-SWE, Karolinska Institutet) have given approval and all participants gave their written informed consent. The study was in accordance with the principles of the current version of the Declaration of Helsinki.

## Results

### Quality control of SNPs and samples and LD pruning

We first removed SNPs that had a genotyping success rate of less than 95% in any of the included studies (Table 2). This led to the removal of all non-autosomal SNPs as only autosomal SNPs were available for the imputed data set. After applying all SNP quality control steps (see methods) there were 184,499 autosomal SNPs available for analysis. Then, 25 samples were removed because they were too closely related to another sample (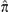
>0.20). Of the remaining samples, 30 with genotype missingness>2% (based on the 184,449 post-QC SNPs) were removed. Of the 184,449 SNPs that passed QC 49,257 remained after LD pruning. The second round of pruning removed only one additional SNP (see Methods).

**Table 2:** Summary of SNP QC.

QC threshold		Removed SNPs	SNPs removed solely for that reason*
***Starting point***		***760,053*** **	
Hardy-Weinberg equilibrium, p<10^−6^		1,061	45
Genotyping rate<.95	CAHRES	196,644	668
	CAPS	439,509	25,839
	DGI	401,945	6,014
	SCZ-SW	396,702	9,941
	TWINGENE-SW	273,685	1,975
	EIRA	349,059	27,371
Minor allele frequency<.01		8,311	70
Between-study comparison (1-vs-rest comparison), any p<10^−6^		142,899	785
***Remaining***		***184,449***	

*SNPs that were not removed due to any other criterion than the one listed.

**The number of SNPs in the largest study (SCZ-SW). All other numbers refer to this number and not the number of SNPs available in each study.

### Detection of Finnish ancestry

**Figure 1:**
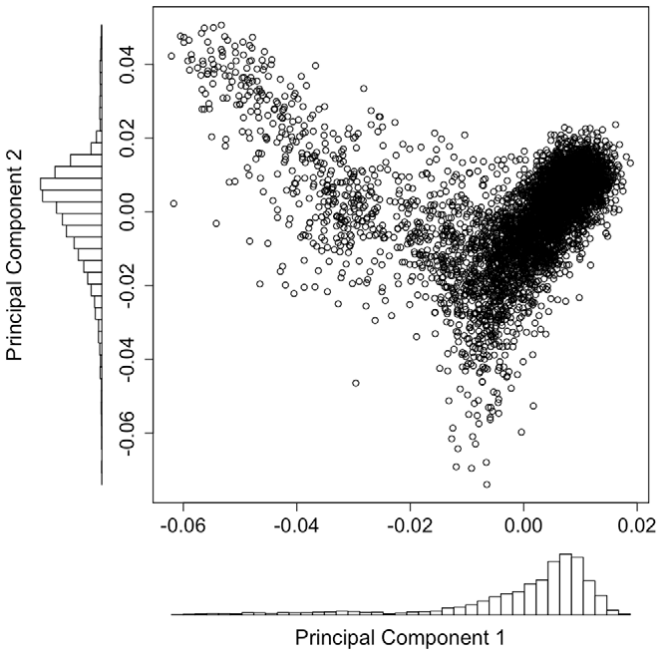
Plot of principal component 1 vs. principal component 2 with corresponding histograms. The histograms show the high density in the main cluster of the plot. The principal components are based on the analysis with 374 samples removed, due to suspected Finnish ancestry and 76 samples removed due to other criteria (see methods and results).

Before having removed any sample due to non-Swedish ancestry we performed a principal component analysis on the genetic data. The two first directions of variation were strongly correlated with Finnish ancestry (defined as having been born in Finland or have had at least one parent or grandparent born there) (Figure S1). With respect to Finnish ancestry, the first two components explained, among the samples for which we had information on ancestry (SCZ-SW, 2,494 of a pre-QC total of 5,624), 58.1% and 58.8% of Nagelkerke's pseudo-R^2^, respectively, and 63.9% when combined. Samples with suspected Finnish ancestry on the basis of their genetic data were then removed (Figure S1). In all, 374 samples (6.7%) were removed due to Finnish ancestry and 76 (1.4%) due to the other criteria (Table S2). After the exclusions of samples we repeated the principal component analysis on the remaining samples (Figure 1).

### Overall population structure

**Figure 2:**
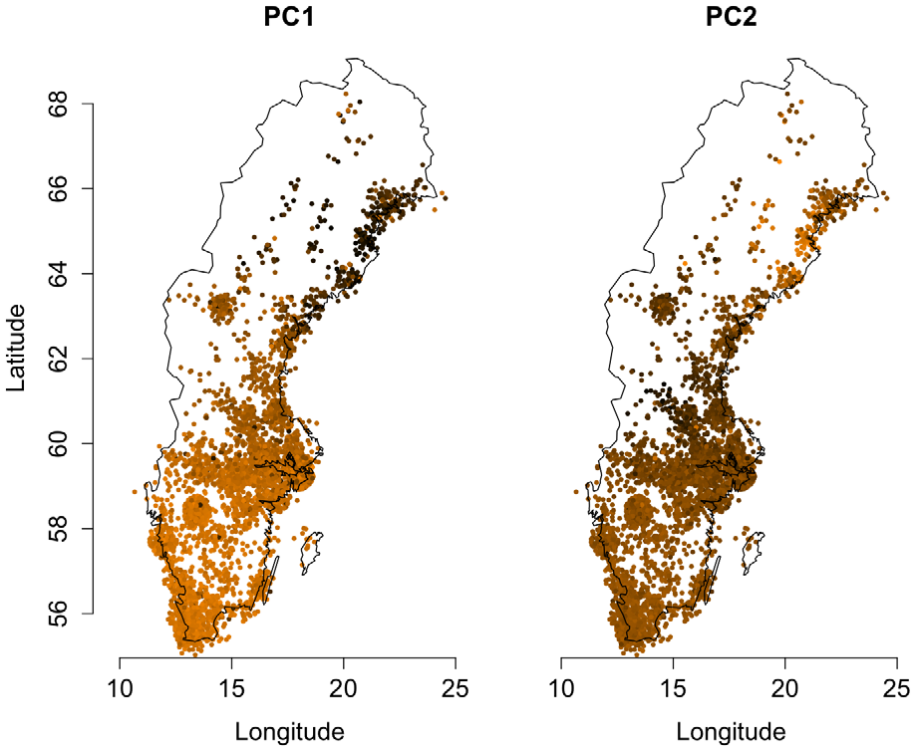
Map plot of principal components 1 and 2. The smallest (large negative) value is represented by dark-brown and the largest positive by yellow. Each sample's location was determined by the best estimate, with birthplace being the first pick (see methods). A small random component was added to each sample's location.

Principal components 1 and 2, from the principal components analysis with samples removed (Figure 1), were the main axes of variation, as indicated by the scree plot (Figure S2). The other principal components had markedly lower eigenvalues. In the plot of components 1 and 2 (Figure 1), most samples fell within one distinct cluster with a small number of samples taking on more extreme values, as visualized by the histograms. Detailed geographical information on each sample allowed us to plot the samples onto a map of Sweden with the value of the principal component determining the color of each sample, with the lowest value represented by dark brown and the highest value represented by yellow (Figure 2). A small random component was added to each coordinate, in order separate samples from the same location that would otherwise have been plotted at the same coordinates and to avoid making individual samples identifiable on the map. The map-based plot of component 1 clearly showed that the component described a north-south gradient with a strong correlation between the principal component and the latitude of each sample (R^2^ = 0.54, *p*<10^−100^). The first component was also associated with longitude (R^2^ = 0.34, *p*<10^−100^), but little additional variance in the component was explained when adding longitude to a model already containing latitude (ΔR^2^ = 0.006) as latitude and longitude were correlated in our sample due to the country's elongation in the southwest-northeast direction.

**Figure 3:**
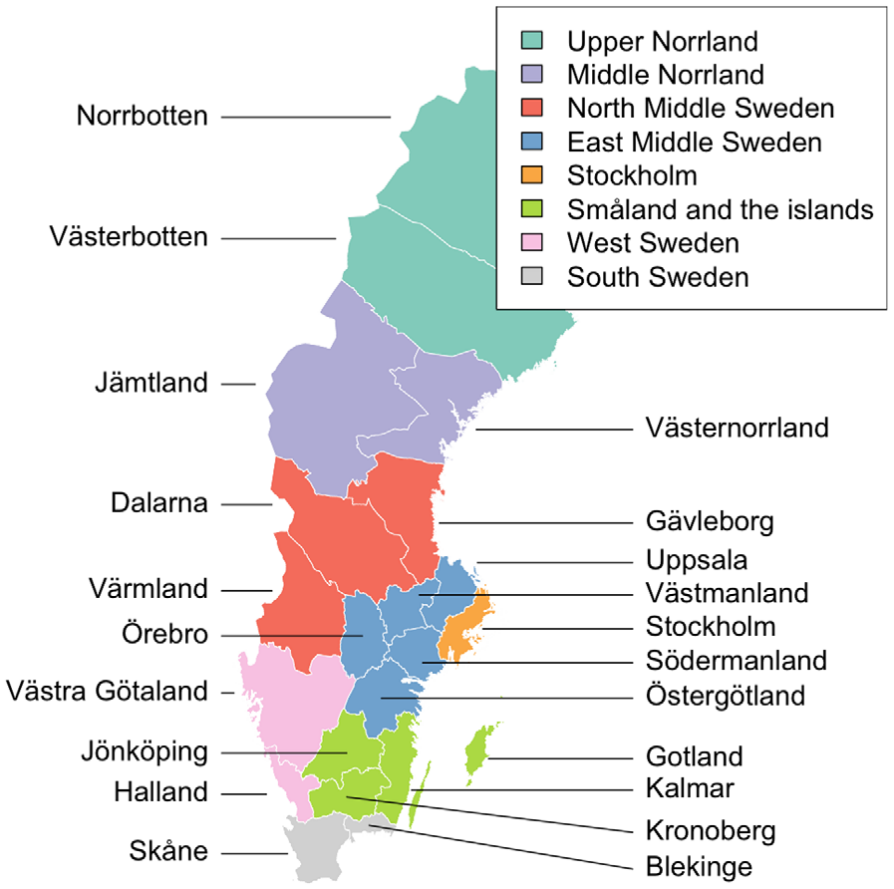
Counties and national areas of Sweden. A national area is comprised of one or more counties. Names of counties are displayed directly on the map while the national areas have been given different colors.

Most of the variability in the first component was due to the more extreme values in the negative direction, which were found in the northernmost counties. Samples from the southern counties take on values much more similar to each other, making up the dense cluster of samples in the plot (Figure 1, see also Figure S3). The 6.9 percent of samples coming from the two northernmost counties of *Norrbotten* and *Västerbotten* together accounted for 52 percent of the variance in principal component 1 (Figure 3).

Novembre and Stephens [26] have demonstrated that highly structured patterns arise as mathematical artifacts when PCA is applied to simulated spatial data in which similarity decays as a function of geographical distance. In their simulation studies the first two principal components corresponded to perpendicular gradients. Although our first principal component described a north-south gradient, our second principal component failed to show a perpendicular/east-west gradient, possibly as a reflection of specific migration events and admixture. The second principal component showed clustering of low values in the middle of the country with the north and south taking higher values, with the north being more accentuated than the south. When comparing the mean value of the second principal component among the samples from each county, *Dalarna county*, in mid-western Sweden, neighboring Norway, had a lower mean than all other counties, significantly different from all other counties with *p*<10^−20^ in all comparisons (Figure S3). Interestingly, the principal component showed a geographical direction within the county, with the most negative values found in the northwest, (*p_lat_ = 0.00016, p_long_ = 0.0023*, linear models).

Using the principal component weights calculated from our primary samples, we calculated the second principal component values for 76 Swedes of self-reported full Finnish ancestry (in SCZ-SW). Full Finnish ancestry was defined as being either born in Finland or having had two parents born there. These 76 individuals had been previously removed from our data set. They had predominantly negative values (*p_mean≠0_* = 2.8×10^−44^) (Figure S4), i.e. values tending in the same direction as the extreme values seen in *Dalarna county*. The values were not of the same magnitude as the most extreme values of individuals in *Dalarna county*, but were consistently negative, indicating that remote Finnish ancestry may be part of the genetic structure detected by the component. We also applied the component to an independent sample of 939 controls collected in Finland; the values for these controls also tended to be negative (*p_mean≠0_*<10^−100^) (Figure S5).

We then applied the weights of the second principal component to a set of 388 Norwegian control samples from the capital Oslo and the neighboring region Akershus. These samples also had values tending in the same direction as the samples from northwestern *Dalarna county* and the Finns (*p_mean≠0_*<10^−100^), In general the values were of smaller magnitude than the component values of the Finnish samples, although some subjects were negative outliers (Figure S6), i.e. in the *Dalarna county* direction.

We also wanted to investigate other possible underlying causes of the observed distribution of the second principal component in the primary data set, broken down by geographical position, such as isolation and consecutive allelic drift. If isolated populations have yet to be broken up, we would expect to see more homozygosity among the samples from the isolated populations, due to a smaller effective population size. We tested for association between the second principal component and the inbreeding coefficient, as calculated by the –het command in Plink, in *Dalarna county* only. For samples within *Dalarna county* the second principal component was indeed associated with a degree of inbreeding (*p* = 0.013), in the expected direction; the extreme values found in the north-western parts of the county were associated with higher degrees of inbreeding.

None of the other principal components showed any geographical clustering which was as distinct as for the first two components (Figure S7).

We also performed an admixture analysis using *Dalarna county* samples, comparing models based on 1 to 5 ancestral populations. The single population solution had the lowest cross validation error.

### Population differentiation with respect to pairwise F_st_

The population pairwise F_st_ was calculated by comparing both (21) counties and (8) national areas (groups of counties) (Figure 3) according to their level 2 and 3 NUTS (Nomenclature of units for Territorial Statistics – the geocode standard for referencing subdivisions of countries, regulated by the European Union) classifications [27]. *Gotland county* was excluded as only 12 samples were available. All other counties had a post-QC minimum of 65 samples. In addition, 76 samples collected in Sweden, but with full Finnish ancestry, were included. We also included the 60 HapMap CEU founders collected in the U.S., of Northern European ancestry, in order to yield a reference with which to compare the F_st_ values observed for the Swedish national areas and counties.

As expected, ethnic Finns showed the largest genetic distances to the other groups, both to the HapMap CEU samples and to the Swedish national areas with F_st_ values ranging from 0.0037 for Stockholm to 0.0053 for the HapMap CEU founders (Table 3). Pairwise F_st_ values were small for all combinations of counties in the southern half of Sweden, consistent with a greater rate of gene flow and reduced level of isolation. F_st_ values were larger for pairs of counties in the northern half of Sweden and for pairs of counties which were in different halves of the country. The northernmost national area of *Upper Norrland* showed considerable differences to all other regions, even to the immediately southern national area of *Middle Norrland* (F_st_ = 0.0011).

**Table 3:** F_st_ values and λs for a fully stratified study of 500 cases and 500 controls between national areas.

	HapMap CEU	Southern Sweden	Småland with the islands	Western Sweden	Stockholm	East Middle Sweden	North Middle Sweden	Middle Norrland	Upper Norrland	Finns
HapMap CEU		0.000545	0.000673	0.000627	0.000585	0.000672	**0.001018**	**0.001309**	**0.002587**	**0.005320**
Southern Sweden	1.55		0.000158	0.000225	0.000180	0.000237	0.000574	**0.000906**	**0.002187**	**0.004633**
Småland with the islands	1.67	1.16		0.000179	0.000112	0.000164	0.000494	**0.000814**	**0.002063**	**0.004318**
Western Sweden	1.63	1.23	1.18		0.000160	0.000215	0.000500	**0.000842**	**0.002112**	**0.004599**
Stockholm	1.59	1.18	1.11	1.16		0.000022	0.000227	0.000496	**0.001686**	**0.003808**
East Middle Sweden	1.67	1.24	1.16	1.22	1.02		0.000195	0.000512	**0.001704**	**0.003661**
North Middle Sweden	**2.02**	1.57	1.49	1.50	1.23	1.20		0.000448	**0.001674**	**0.003665**
Middle Norrland	**2.31**	**1.91**	**1.81**	**1.84**	1.50	1.51	1.45		**0.001146**	**0.003855**
Upper Norrland	**3.59**	**3.19**	**3.06**	**3.11**	**2.69**	**2.70**	**2.67**	**2.15**		**0.004613**
Finns	**6.32**	**5.63**	**5.32**	**5.60**	**4.81**	**4.66**	**4.67**	**4.86**	**5.61**	

National areas are sorted south-north with the southern ones at the top of the table. F_st_ values above the diagonal, λs below. Comparisons with F_st_>0.0008 or E(λ)>1.8 have been made bold.

The southern national areas were closer to the HapMap CEU samples than to the northernmost Swedish national areas, especially *Upper Norrland*. This was also true when the national areas were split into individual counties (Table S3).

**Figure 4:**
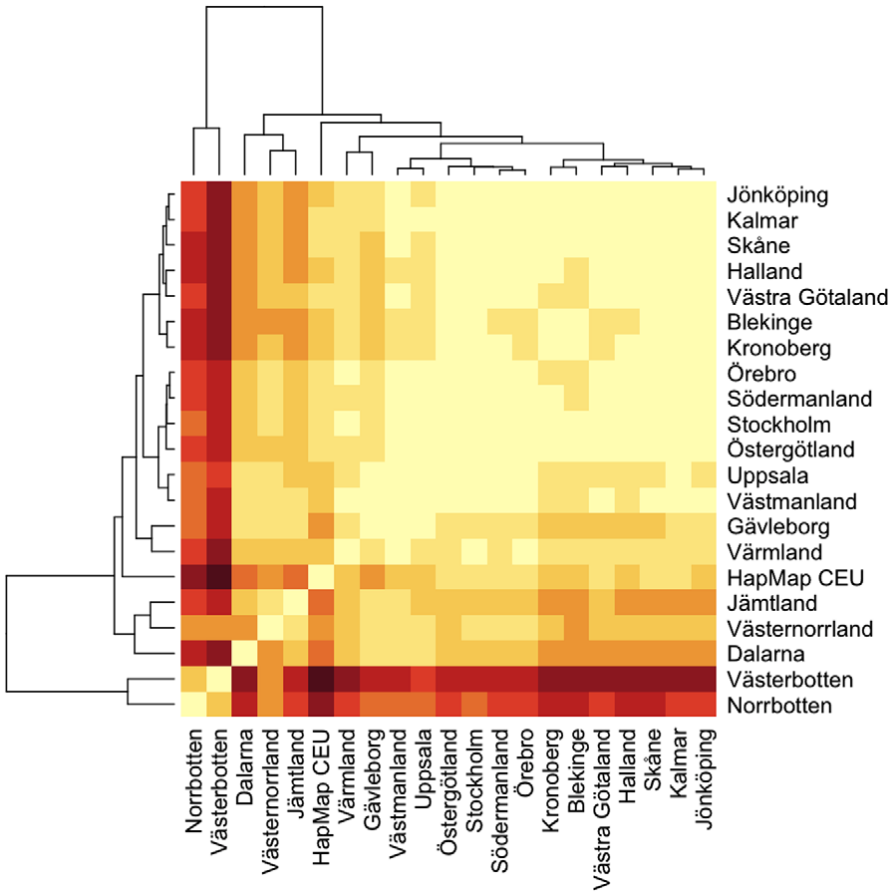
Heat map with hierarchical clustering of counties based on F_st_.

We visualized the F_st_ values between individual counties together with the HapMap CEU samples using a heat map, with the counties ordered according to a hierarchical clustering algorithm (as implemented in *hclust* in R) and using a multidimensional scaling (MDS) plot. Because Finns have the largest difference from other groups, they were excluded from these plots - to avoid their overshadowing of differences between the other groups. The heat map (Figure 4) shows that the northern counties were more different from each other than the southern counties were from each other. According to the dendogram obtained from the hierarchical cluster analysis, the northern counties were grouped into a larger number of clusters. For example, if we define a 7 cluster solution from the hierarchical tree, the clusters are defined as (i) *Norrbotten*, (ii) *Västerbotten*, (iii) *Västernorrland* and *Jämtland*, (iv) *Dalarna*, (v) *Värmland* and *Gävleborg*, (vi) HapMap CEU samples and (vii) all other Swedish counties. The MDS plot also clearly demonstrates that the northern counties are more clearly genetically differentiated from each other than the southern counties are from each other, and highlights also that Dalarna is differentiated from other Swedish counties (Figure S8).

In order to assess whether any confounding was arising from genotyping platform differences and sample collection methods we repeated the F_st_ calculations on the national areas using the largest sample set SCZ-SW (Table S4). This decreased the geographical coverage, especially in the northern counties and national areas. The results were consistent with the ones derived from all sample sets, but the differences between the northernmost national areas and the rest of the sample decreased somewhat, with the largest observed between-national area F_st_ value declining from 0.0022 to 0.0017.

We also calculated between source study F_st_ to assess possible bias due to genotyping platform, sample collection etc., and ran two separate analyses in *Skåne* and *Stockholm counties* for which a large number of samples were available in most studies. The differences between the studies within each of the counties were generally small but in some cases significant, with a mean F_st_ of 0.00013 and 0.00008 for *Skåne* and *Stockholm county* respectively (Table S5). The maximum F_st_ observed between the studies were 0.00026 and 0.00020 respectively, much lower than most of the F_st_ values observed between the national areas and counties in the main analysis. As the source studies had different types of geographical information on the controls (birth or residence) some differences are to be expected. Also, the studies may have collected samples in a more limited geographical region than the entire county, which may create differences when comparing samples from different studies.

In order to assess the effect of the observed stratification on power loss in case-control studies, we carried out simulations (Figure S9). For a SNP with a minor allele frequency of 0.16 and an odds ratio of 2, a case-control study of 500 cases and 500 controls would have about 80% power of detecting association, based on using a genome-wide significance threshold of 5×10^−8^ (if the sample was selected from a population without stratification). The power would be reduced to less than 20% if the genomic inflation λ was 2. If the minor allele frequency instead was 0.1 the power to detect association would be 40% in an unstratified data set but almost none if λ was 2. The values of λ which we observed for pairs of national areas within Sweden ranged from close to 1 to more than 3, for the most disadvantageous pair of national areas.

### Extended regions of homozygosity

We finally examined extended regions of homozygosity in the samples. We chose to look for extended regions instead of calculating an inbreeding coefficient based on all SNPs as imputation may bias the results. We did observe some differences in the mean number of extended homozygous segments between the studies (not shown). This does not necessarily need to have arisen due to technical artifacts but may be due to the geographical differences in sampling. However, as differences were observed, we chose to control for the sample set when comparing the number of segments between different counties.

**Figure 5:**
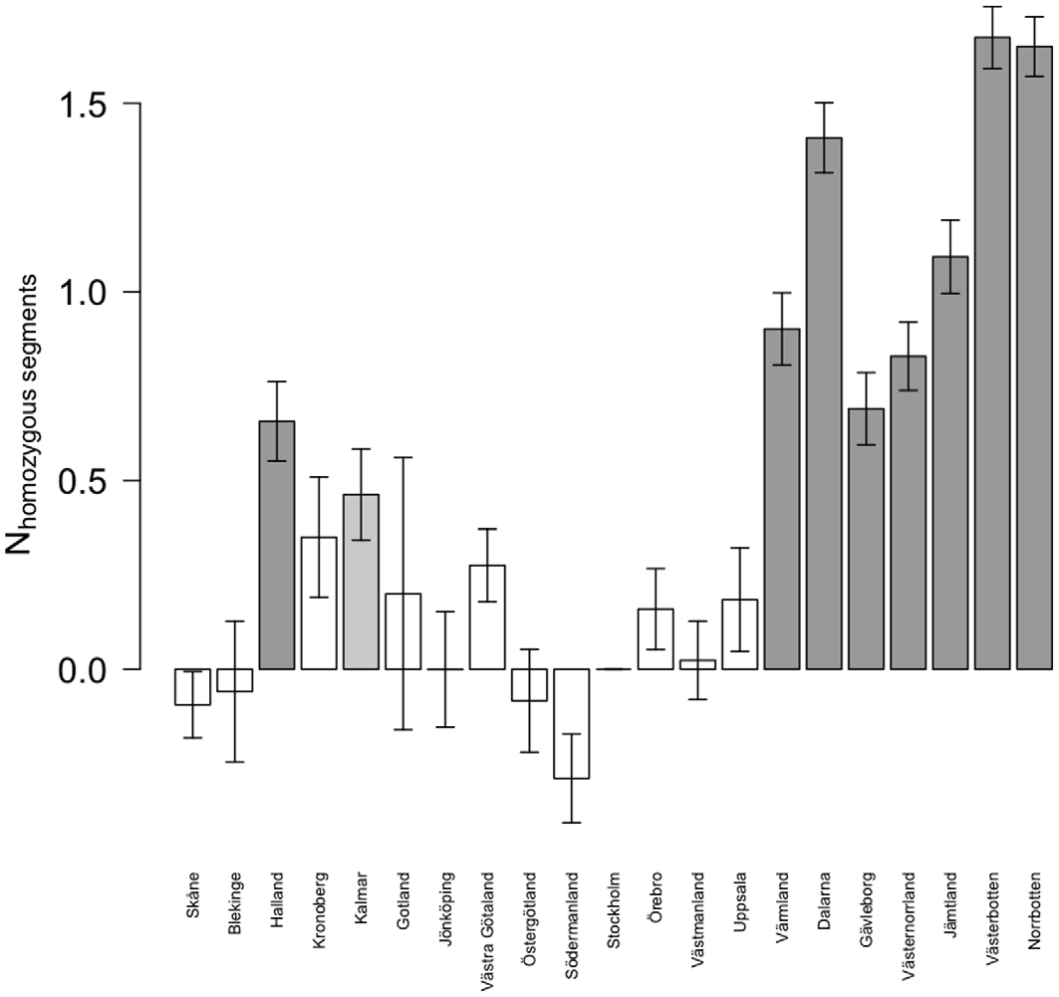
Poisson model of the mean number of homozygous segments in each county. Bar chart of estimates of regression coefficients, with standard error bars. Model uses *Stockholm county* as baseline and adjusts for source study. Dark gray bars p<10^−5^, light gray bars p<0.05, white bars p> = 0.05 (with p values adjusted for the multiple testing of 20 counties). Counties are sorted south to north.

The number of homozygous segments increased by 0.20 for each degree of latitude (*p*<10^−100^) in a Poisson model where we controlled for sample study. In addition, we fitted a model with county instead of latitude as a covariate, with *Stockholm county* as baseline, controlling for sample study (Figure 5). The county-specific analysis showed that the samples from the northern counties generally had a larger number of homozygous segments, consistent with a reduction in genetic diversity. Two of the southern counties (*Halland* and *Kalmar counties*) also showed significant increases in the number of extended homozygous segments.

## Discussion

This study was conducted both to investigate the extent of genetic stratification within Sweden and to aid future population-genetic sample collections. In order to get a set of samples covering the entire country, we combined controls from different studies, resulting in a post-QC set of 5,174 Swedish controls.

### Patterns of the principal components

The first principal component showed the presence of a north-south genetic gradient that was mainly driven by each northern county being different from other counties. Systematic variation was present in the south of Sweden although to a markedly smaller degree than in the north of Sweden. Even though the samples from the northern counties made up a small part of our data set, consistent with the distribution of the Swedish population, they were responsible for half the variance in the principal component. The overall North-South axis of variation is consistent with previous studies that have shown axes of variation on a European scale that closely line up with geographical axes [4], [5], [11], [28], [29].

The second principal component did not display a constant gradient across Sweden. The county with the lowest mean was *Dalarna*, with samples in the north-western parts of this county contributing with particularly low values. These patterns may be linked to Finnish or Norwegian ancestry, although the component most probably encompasses other signals arising from factors such as isolation and drift in the more remote parts of *Dalarna county*. A large wave of migration did take place from parts of today's Finland to *Dalarna, Värmland and Gävleborg counties* during the 16^th^ and 17^th^ centuries, with some settlers moving further west to Norway. The migrants, known as the “Forest Finns”, have today become fully assimilated into Norwegian and Swedish society with almost none speaking Finnish. As we conservatively removed samples that had Finnish ancestry to reduce the effect of ethnic Finns on the Swedish population stratification, we removed samples that had a substantial Finnish contribution. Indeed, the samples that had the most extreme values with respect to principal component 2 fell close to our cutoff used to define Finnish ancestry (Figure S10). What we see may therefore be the remnants of early Finnish migrations to Sweden. It could be that even after the removal of samples with Finnish ancestry, Finnish influx leaves its mark, consistent with a long history of Finnish migration to Sweden coupled with a high degree of admixture. However, when we applied the component to a set of Norwegian samples, they too had negative component values with a small number of samples being clear outliers in the negative direction (Figure S6). It is therefore possible that a portion of the component predicts Norwegian ancestry, or isolation and drift in the border areas that has left its mark in both populations, or a combination of these factors.

### Population pairwise F_st_ values

The observed genetic differences between the southern national areas and counties are similar to what has been seen in the UK, when comparing southern England to Scotland, and the genetic differences between the southern national areas and *North Middle Sweden* (the approximate geographical center of the country) are similar to what has been observed between the UK and Ireland [9]. They are much smaller than the ones observed between regions in Finland where the F_st_ has been shown to be about an order of magnitude larger than what we observed among the southern Swedish national areas [7]. The genetic differences between *Upper Norrland*, the northernmost national area, and the southern and middle national areas were much larger than the genetic distances we observed between the southern national areas and the HapMap CEU founders, highlighting the extent of the distances. The genetic differences observed between the southern national areas and the northernmost one were larger than what has previously been observed between Swedes and Danes, and Swedes and the Dutch [5]. This puts the observed intra-Sweden genetic differentiation into perspective, showing that the genetic differences between the southern and northern parts of Sweden are of larger magnitude than those between a general sample of Swedes and samples from some other European countries and Americans of Northern European ancestry.

In a genetic association study, the expected genomic inflation λ values that would arise if a stratified data set of 500 cases and 500 controls from different populations was used, indicate that less stratification would be present if the HapMap CEU founder population was compared against a sample from the southern national areas of Sweden than if the southern national areas were compared against the northernmost. Americans of northern European ancestry (in the CEU sample) are not generally considered to be suitable controls for Swedish cases, and have not been used in any genomewide asssociation scan of Swedish cases. Geographically poorly matched Swedish controls may be equally unsuitable.

According to our estimates, a genomic inflation λ of 2 or more would be possible by sampling cases and controls in different counties in Sweden, illustrating the need to geographically match cases and controls in a study. Without having information on a genome-wide panel of SNPs, stratification would be hard to detect if only data on a small number of typed SNPs were available, making false-positives generated by genetic stratification a clear possibility. Having a genome-wide coverage of SNPs would rather result in a substantial power loss and not false positives, assuming the genomic inflation λ would be used to control for the stratification. Even if case and control sampling locations did overlap to some degree, genetic stratification would still exist, but to a lesser extent.

### Extended homozygous segments

The observed patterns of homozygosity further reinforce the picture of a genetic divide between the southern and northern parts of Sweden. While the differences in the number of extended homozygous segments were mostly small and non-significant for the southern national areas, the counties corresponding to the three northernmost national areas had a larger number of extended homozygous regions, suggestive of an increase in autozygosity. The northern national areas are much more sparsely populated than the southern with vast geographical distances between towns and villages, a fact that may have contributed to the observed loss of heterozygosity. The increased level of genetic homogeneity in the north may be an advantage if a study is set to search for recessive genetic variants with high penetrance, as by means of linkage studies.

### Concluding remarks

The main south-north pattern detected in the principal component analysis may be the amalgamation of many separate factors. Immigration of continental Europeans, and Britons to the southern parts of Sweden may be one such factor, together with isolation and consecutive allelic drift and admixture with the indigenous Sami people in northern Sweden.

Our results show that even a study of modest size, but with poorly matched geographical distributions of cases and controls in Sweden, e.g. with cases from the north and controls from the south, would suffer a drastic increased type 1 or type 2 error rates (depending on whether stratification could be controlled for or not). The southern and middle parts of Sweden are more genetically homogenous, contributing only a small part to the variation in the first principal component and showing small population pairwise F_st_. They do in general show less extended homozygosity than the three northernmost national areas. As indicated by both the first principal component and the table of F_st_ values, a gradient exists along the length of the country, with most of the variation driven by samples from the northernmost counties. The similarities between the southern counties and national areas make them suited for population-genetic sample collection efforts, as shown by the pairwise F_st_.

The picture of the Swedish genetic structure presented here can only be considered as a historical snapshot. Immigration and movements around Sweden can alter the picture we have described considerably within the span of only a few generations. Within country population movement creates genetic diversity and breaks up existing structures. Immigration from other countries leads to new stratification of a vastly complex nature. With increased movements, homozygosity, due to a high degree of kinship with geographically local possible partners, may swiftly decrease.

When conducting a population-based case-control study, it is essential to obtain samples of cases and controls that are genetically similar. Geographical differences between case and control groups may obscure true genetic associations. We have shown that caution must be taken if Swedes from the northernmost counties are included in a population-based genetic study, since they differ genetically from southern Swedes and also amongst themselves. Genetic patterns do not necessarily follow national borders and substantial genetic differences may be detectable within a country.

## Supporting Information

Figure S1 **Finnish ancestry principal component cutoff.** Principal components stem from the first principal component analysis, which was performed prior to the removal of samples with Finnish ancestry.
(PDF)

Figure S2 **Scree plot (plot of eigenvalues vs rank) of components 1 to 25 from the principal component analysis with individuals of suspected Finnish descent removed.** The PCA was performed after the removal of samples with suspected Finnish ancestry that in turn was based on the first two components from the PCA with individuals of Finnish descent included.
(PDF)

Figure S3 **Principal components by county.** Principal components stem from the principal component analysis performed after the removal of samples with suspected Finnish ancestry.
(PDF)

Figure S4 **Histogram of PC2 values in 76 Swedes of Finnish ancestry (collected as part of the SCZ-SW study).** The samples were not included in the derivation of PC2 (PCA after removal of samples with Finnish ancestry), rather the component was calculated by applying the allelic weights for each individual SNP and then summing over all alleles for each sample.
(PDF)

Figure S5 **Histogram of PC2 values for 939 Finns from a separate study.** The samples were not included in the derivation of PC2 (PCA after removal of samples with Finnish ancestry) rather the component was calculated by applying the allelic weights for each individual SNP and then summing over all alleles for each sample.
(PDF)

Figure S6 **Histogram of PC2 values for 388 Norwegians from a separate study.** The samples were not included in the derivation of PC2 (PCA after removal of samples with Finnish ancestry), rather the component was calculated by applying the allelic weights for each individual SNP and then summing over all alleles for each sample.
(PDF)

Figure S7 **Map plots of principal components 3 to 10.** Principal components illustrated by colors on map with the most negative value as yellow and the most positive as black. Principal components stem from the principal component analysis, performed after the removal of samples with Finnish ancestry.
(PNG)

Figure S8 **Multidimensional Scaling plot of F_st_ values; counties in Sweden and HapMap CEU samples.**
(PDF)

Figure S9 **Effect of stratification on power loss in a sample of 500 simulated cases and 500 simulated controls, using a genome-wide significance cutoff of 5×10^−8^.** Case and control SNPs were simulated in R using rbinom, the chi-square statistic (1 df) was then calculated. This was repeated for 10 000 replicates to derive a distribution of chi-squares used to calculate power. The chi-squares were then corrected for the different levels of λ and power recalculated with respect to the adjusted chi-squares.
(PDF)

Figure S10 **Small PC2 values close to Finnish cutoff.** Samples in the lowest percentile of PC2 (PCA after removal of samples with suspected Finnish ancestry) plotted in red with other samples plotted as either gray (removed due to suspected Finnish ancestry) or black (not removed). Axes are based on the principal component analysis with individuals of Finnish ancestry included.
(PDF)

Table S1 **Swedish population density estimates 2010, per sq. km. by county.** Source: Statistics Sweden (http://www.scb.se).
(DOC)

Table S2 **Samples from each study, the number removed and the number remaining.**
(DOC)

Table S3 **F_st_s and λ_GC 1000_s between counties (excluding Gotland county).**
(DOC)

Table S4 **F_st_s and λ_GC 1000_s between national areas, using data from the SCZ-SW study only.** Differences above 0.008 (F_st_) or 1.8 (λ_GC 1000_) have been made bold.
(DOC)

Table S5 **Between-study F_st_ comparison. Stockholm county above and Skåne county below.**
(DOC)
