# Supplementary figures and images for: The Genetic Structure of the Swedish Population
Source: PLoS One. 2011 Aug 4;6(8):e22547. doi: 10.1371/journal.pone.0022547 (PMC3150368; doi:10.1371/journal.pone.0022547)

## Finnish ancestry cutoff

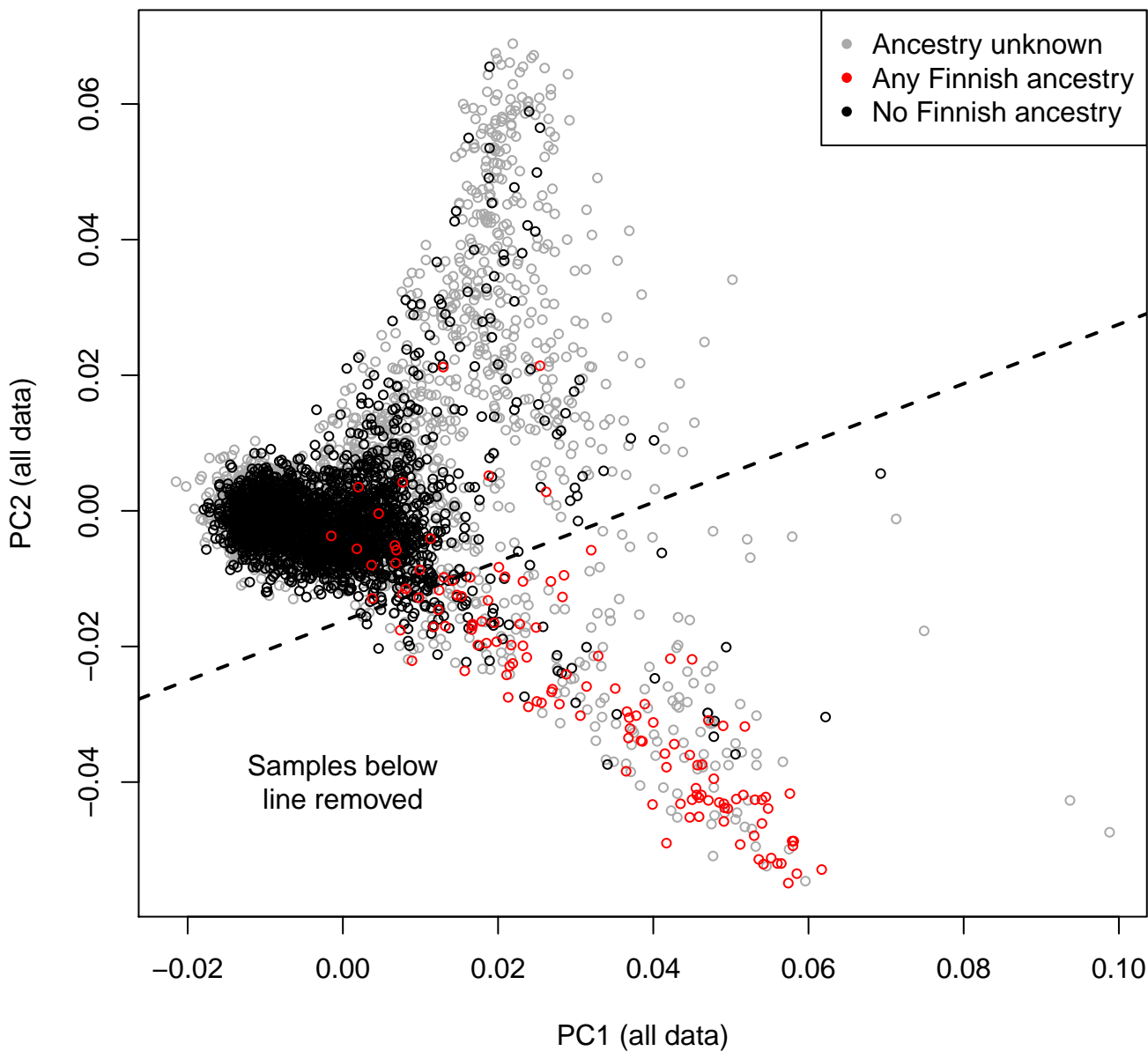

Supplement: Figure S1 — Finnish ancestry principal component cutoff. Principal components stem from the first principal component analysis, which was performed prior to the removal of samples with Finnish ancestry. (PDF) [file pone.0022547.s001.pdf]

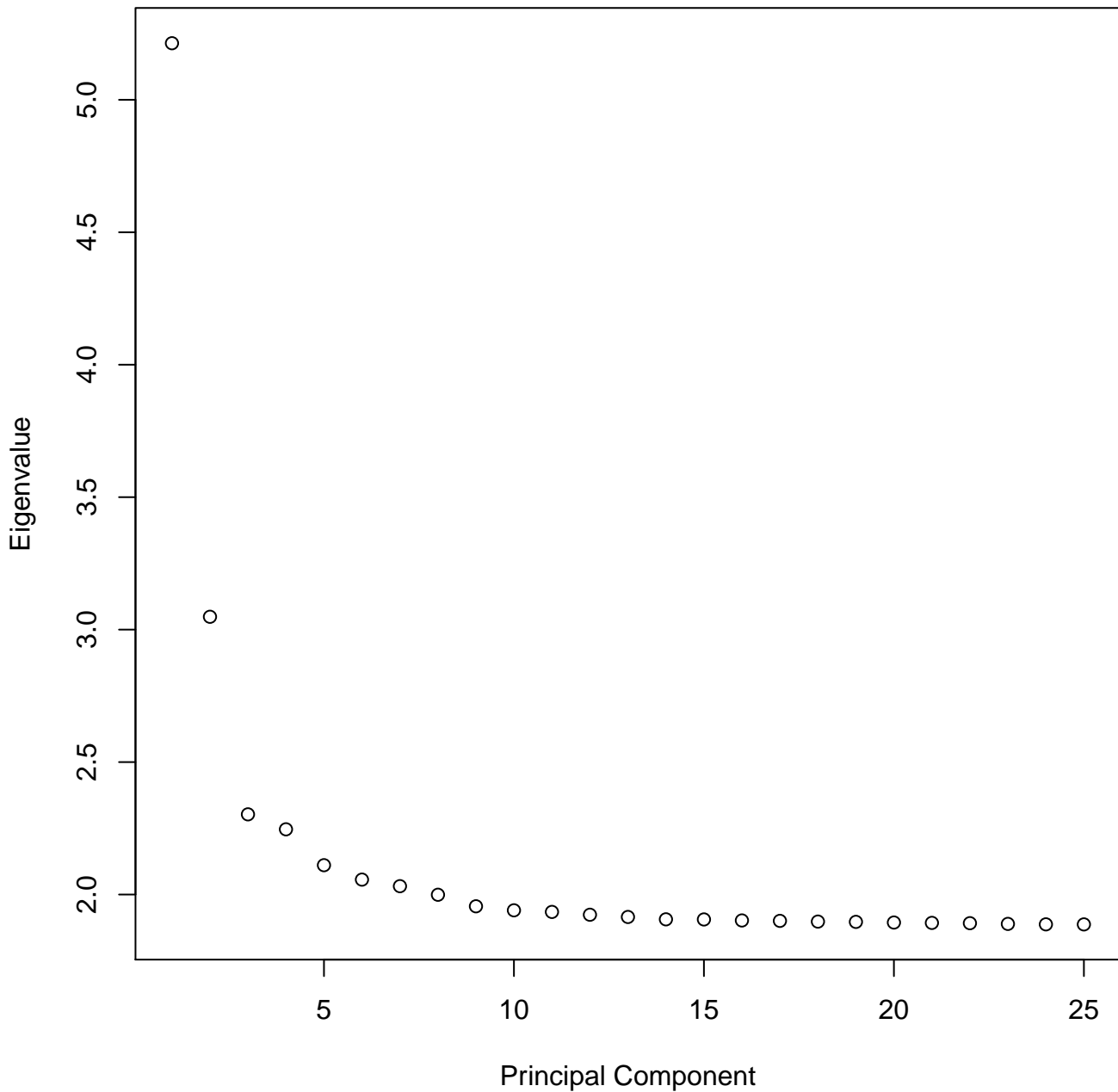

Supplement: Figure S2 — Scree plot (plot of eigenvalues vs rank) of components 1 to 25 from the principal component analysis with individuals of suspected Finnish descent removed. The PCA was performed after the removal of samples with suspected Finnish ancestry that in turn was based on the first two components from the PCA with individuals of Finnish descent included. (PDF) [file pone.0022547.s002.pdf]

PC1

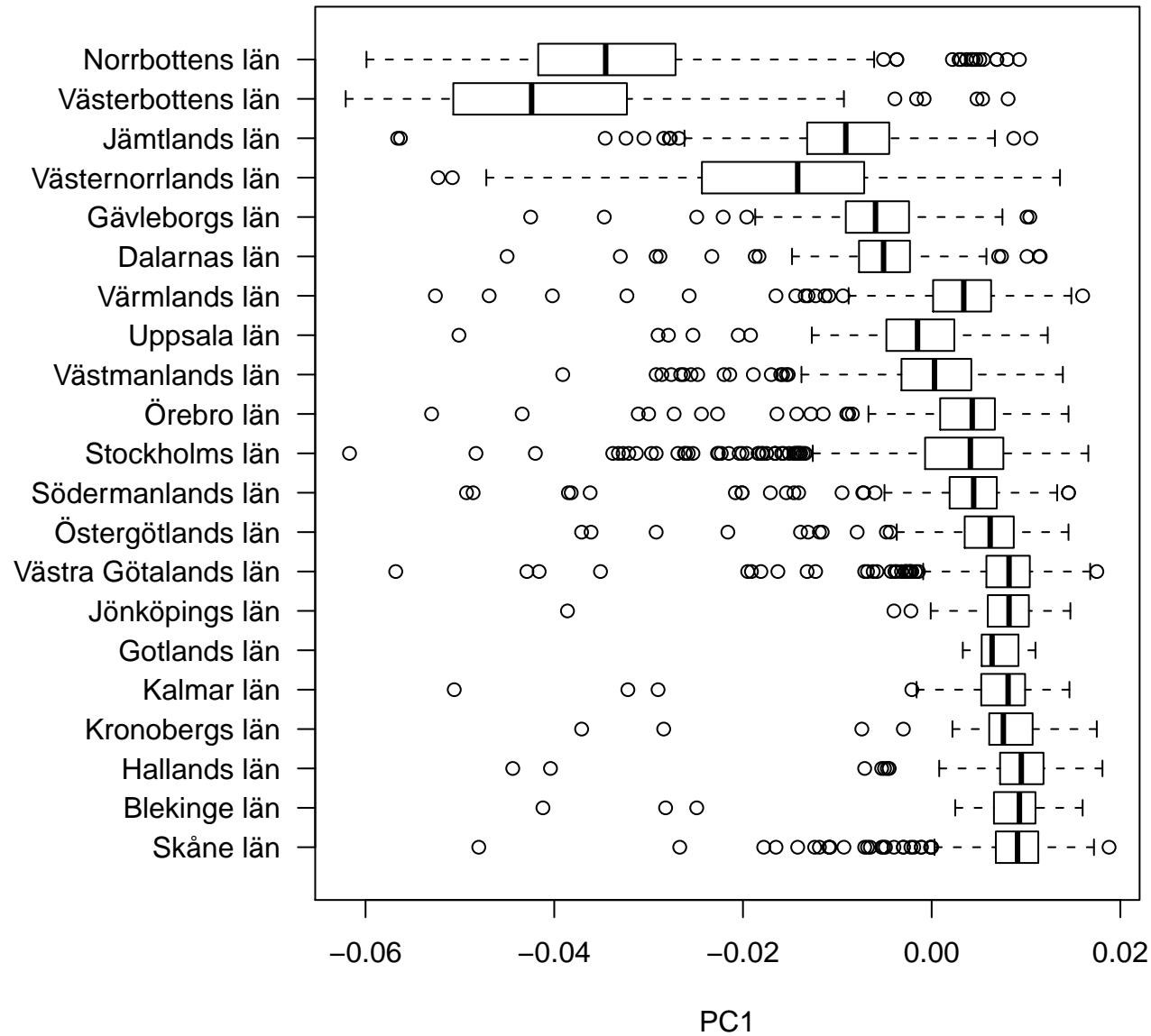

PC2

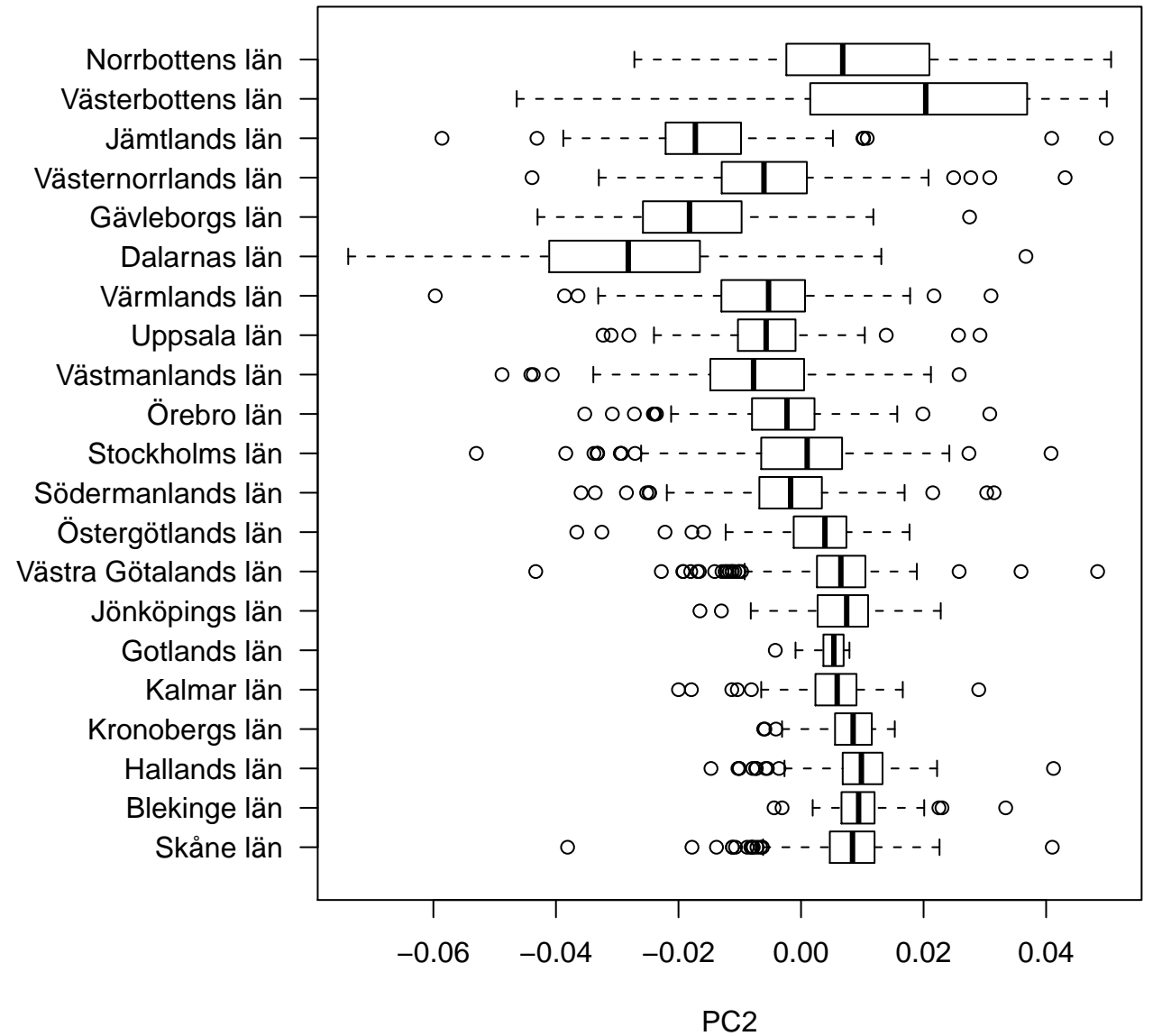

Supplement: Figure S3 — Principal components by county. Principal components stem from the principal component analysis performed after the removal of samples with suspected Finnish ancestry. (PDF) [file pone.0022547.s003.pdf]

**Histogram of PC2 in 76 Finns**

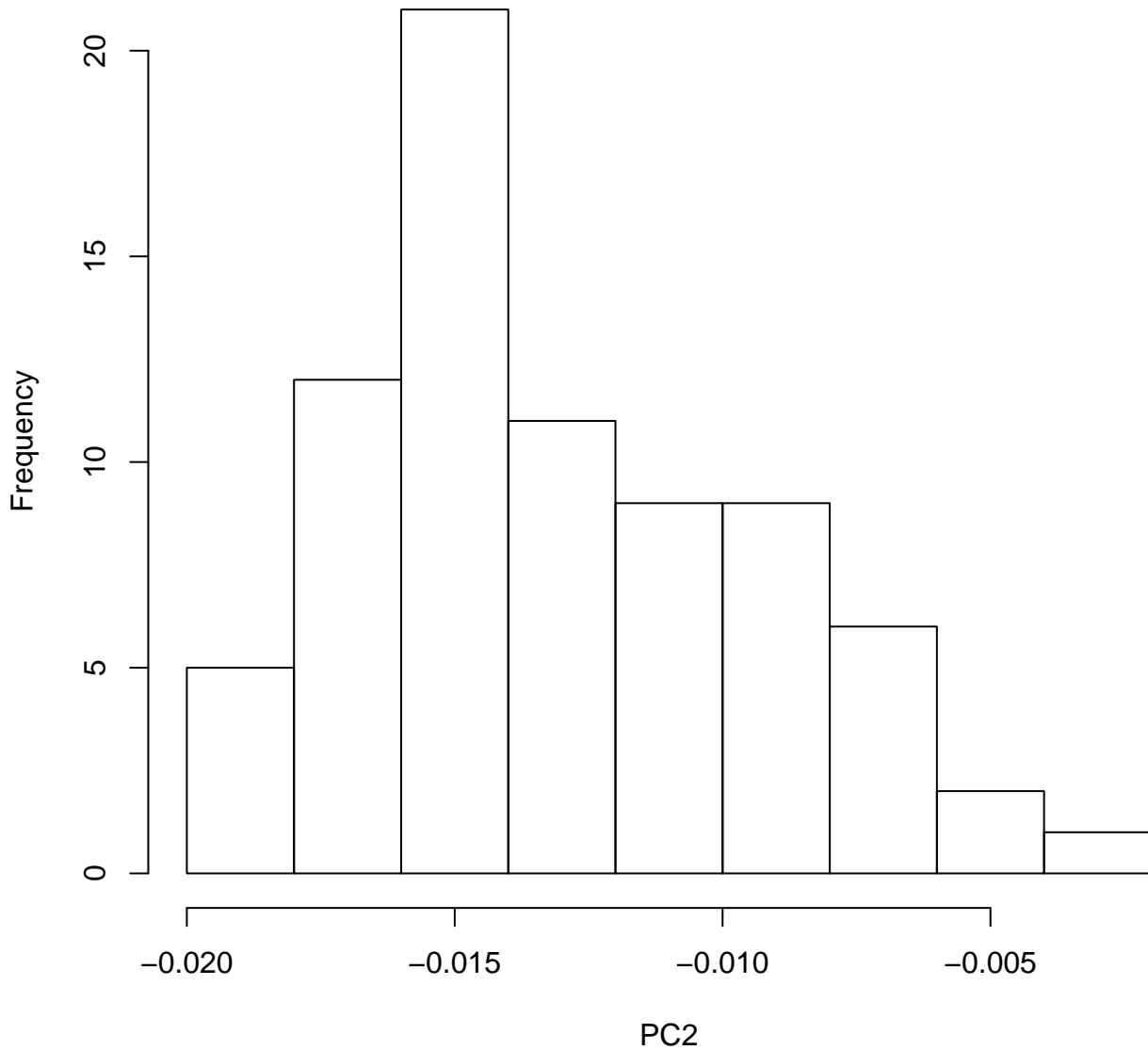

Supplement: Figure S4 — Histogram of PC2 values in 76 Swedes of Finnish ancestry (collected as part of the SCZ-SW study). The samples were not included in the derivation of PC2 (PCA after removal of samples with Finnish ancestry), rather the component was calculated by applying the allelic weights for each individual SNP and then summing over all alleles for each sample. (PDF) [file pone.0022547.s004.pdf]

**Histogram of PC2 in 939 Finns**

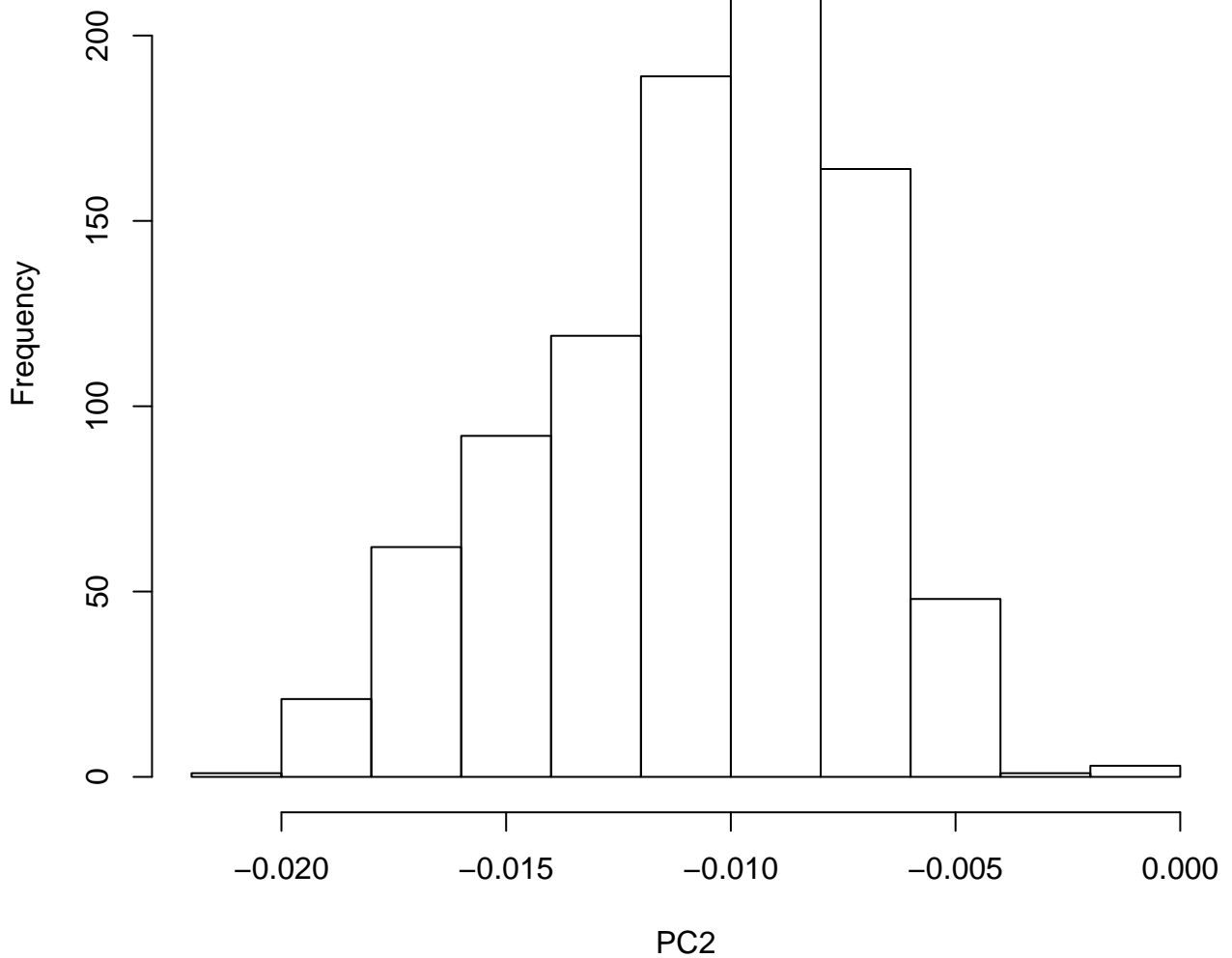

Supplement: Figure S5 — Histogram of PC2 values for 939 Finns from a separate study. The samples were not included in the derivation of PC2 (PCA after removal of samples with Finnish ancestry) rather the component was calculated by applying the allelic weights for each individual SNP and then summing over all alleles for each sample. (PDF) [file pone.0022547.s005.pdf]

**Histogram of PC2 in 388 Norwegians**

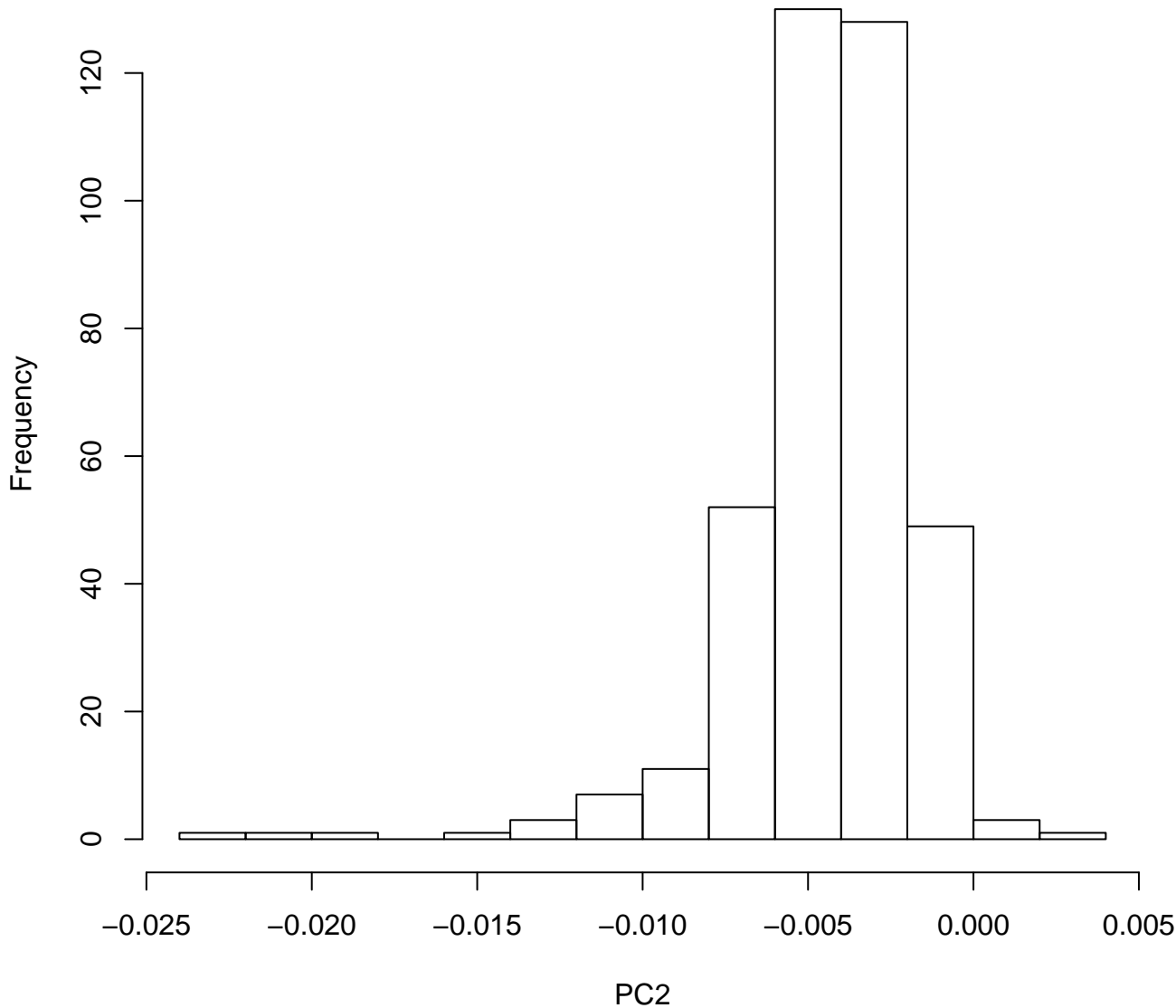

Supplement: Figure S6 — Histogram of PC2 values for 388 Norwegians from a separate study. The samples were not included in the derivation of PC2 (PCA after removal of samples with Finnish ancestry), rather the component was calculated by applying the allelic weights for each individual SNP and then summing over all alleles for each sample. (PDF) [file pone.0022547.s006.pdf]

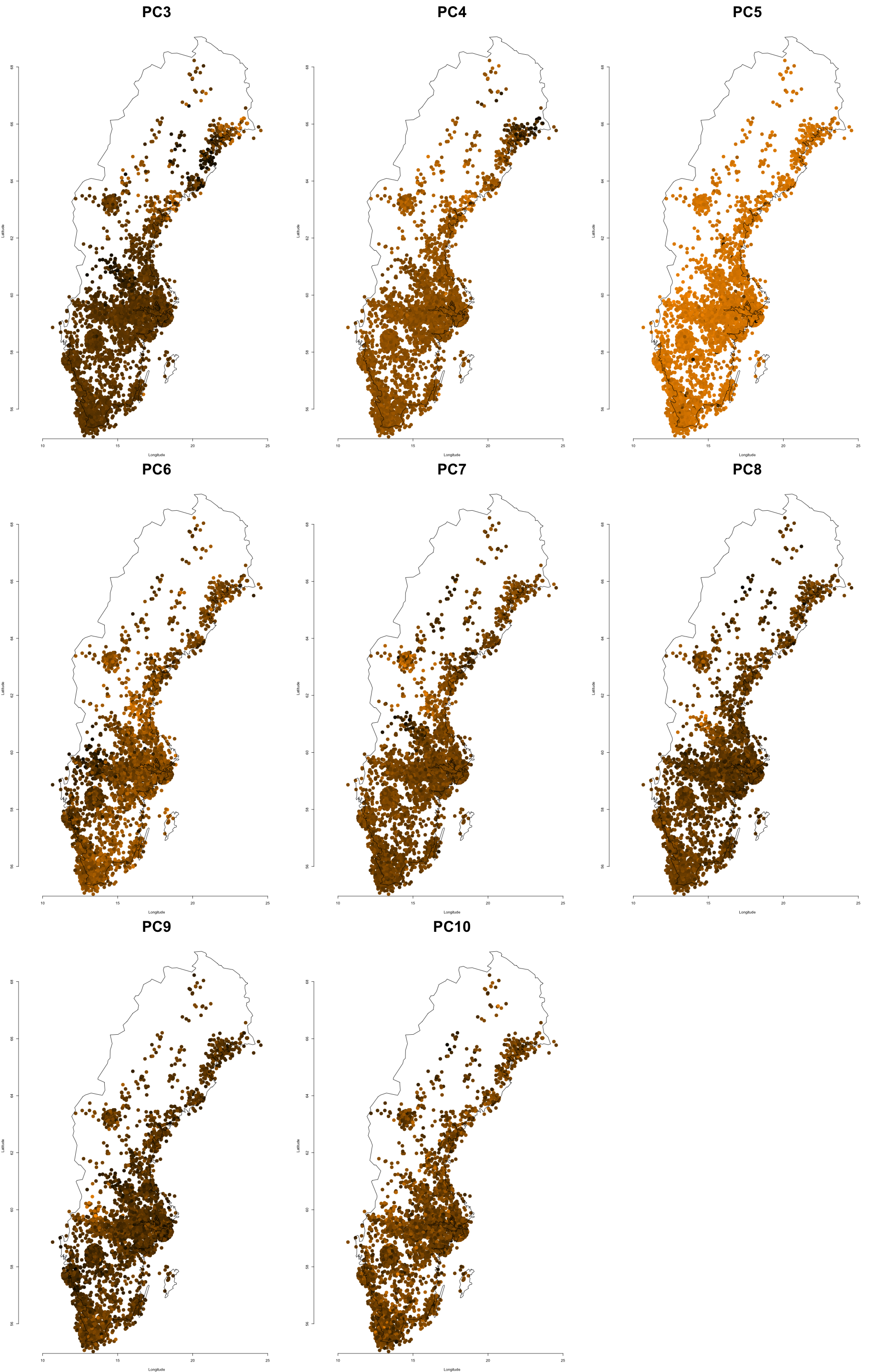

Supplement: Figure S7 — Map plots of principal components 3 to 10. Principal components illustrated by colors on map with the most negative value as yellow and the most positive as black. Principal components stem from the principal component analysis, performed after the removal of samples with Finnish ancestry. (PNG) [file pone.0022547.s007.png]

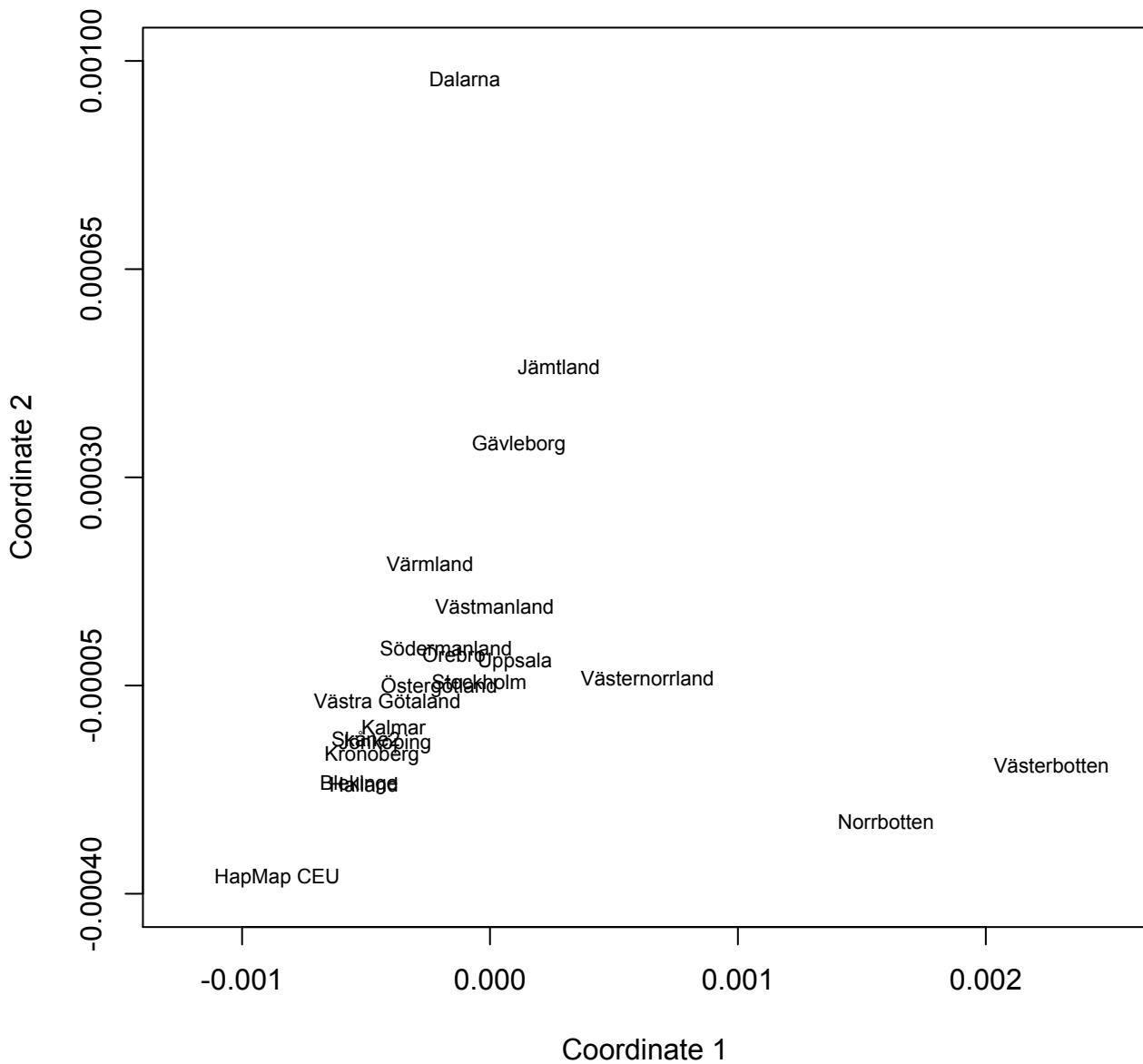

Supplement: Figure S8 — Multidimensional Scaling plot of Fst values; counties in Sweden and HapMap CEU samples. (PDF) [file pone.0022547.s008.pdf]

**Effect of lambda on power**

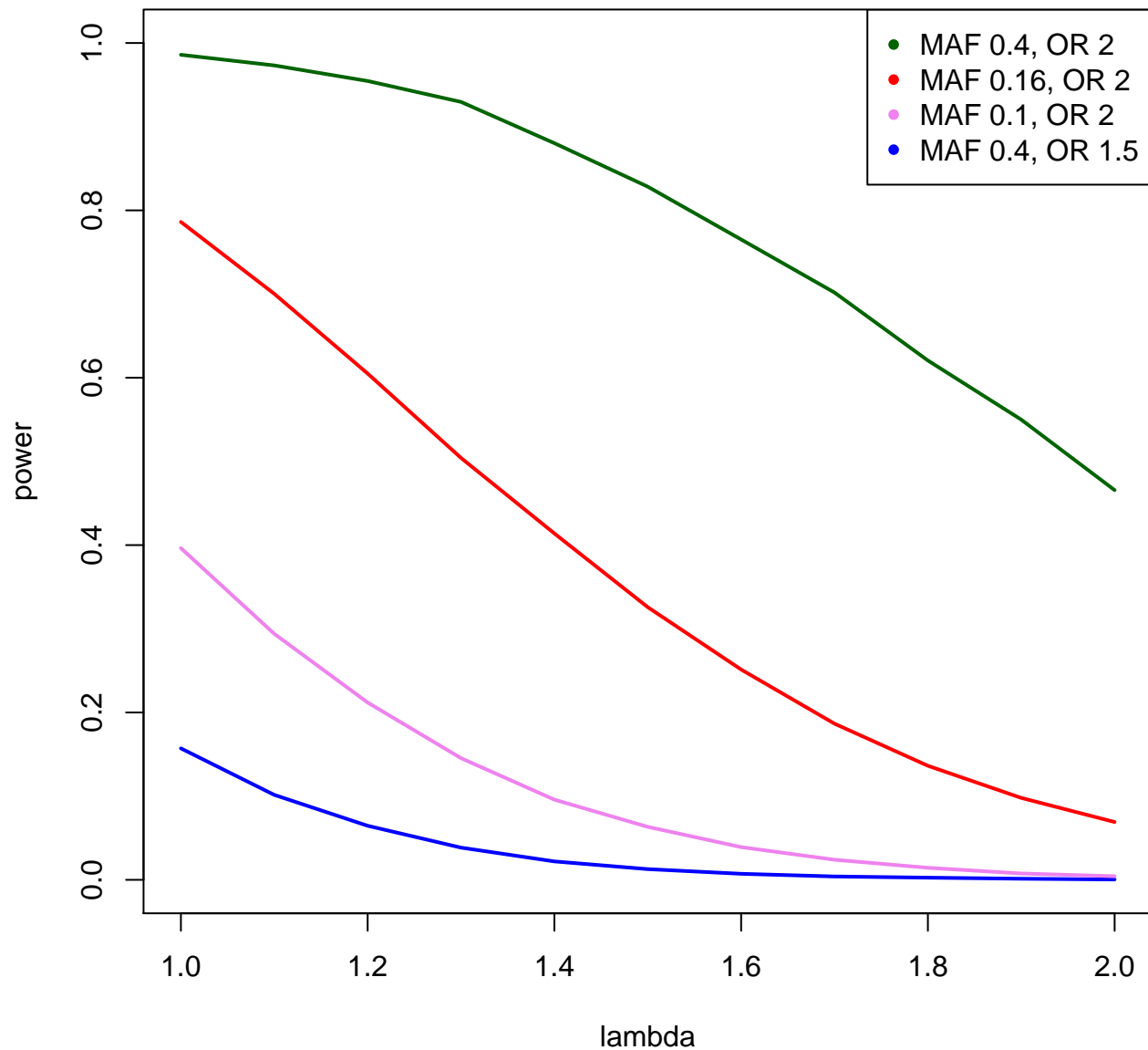

Supplement: Figure S9 — Effect of stratification on power loss in a sample of 500 simulated cases and 500 simulated controls, using a genome-wide significance cutoff of 5×10^−8^. Case and control SNPs were simulated in R using rbinom, the chi-square statistic (1 df) was then calculated. This was repeated for 10 000 replicates to derive a distribution of chi-squares used to calculate power. The chi-squares were then corrected for the different levels of λ and power recalculated with respect to the adjusted chi-squares. (PDF) [file pone.0022547.s009.pdf]

## Small PC2 values close to cutoff

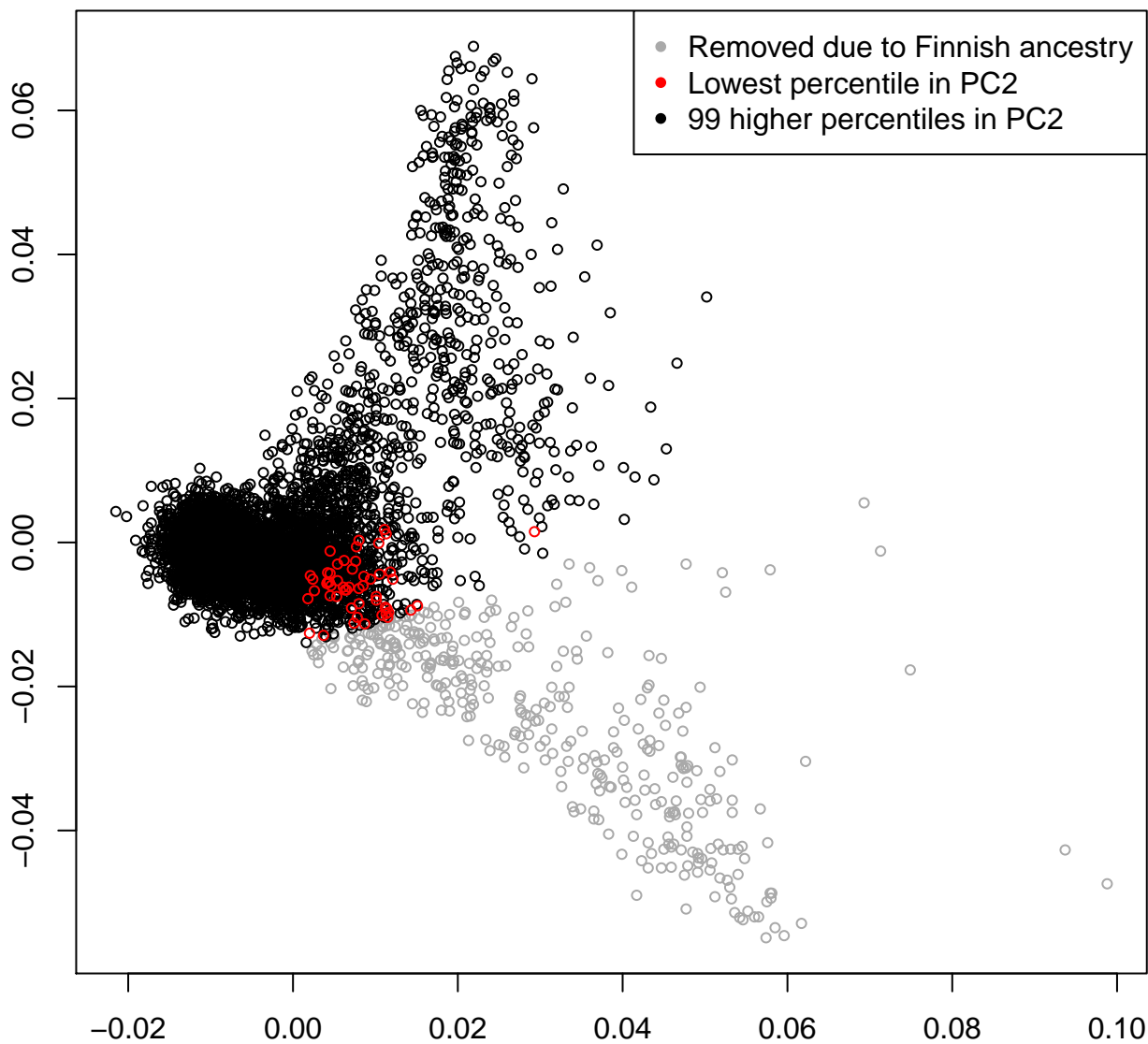

Supplement: Figure S10 — Small PC2 values close to Finnish cutoff. Samples in the lowest percentile of PC2 (PCA after removal of samples with suspected Finnish ancestry) plotted in red with other samples plotted as either gray (removed due to suspected Finnish ancestry) or black (not removed). Axes are based on the principal component analysis with individuals of Finnish ancestry included. (PDF) [file pone.0022547.s010.pdf]
