# Supplementary material for: The Genetic Structure of the Swedish Population
Source: PLoS One. 2011 Aug 4;6(8):e22547. doi: 10.1371/journal.pone.0022547 (PMC3150368; doi:10.1371/journal.pone.0022547)
Supplement: Table S1 — Swedish population density estimates 2010, per sq. km. by county. Source: Statistics Sweden (http://www.scb.se). (DOC) [file pone.0022547.s011.doc]

| County | Population Density per sq. km. |
| --- | --- |
| Stockholm  Uppsala  Södermanland  Östergötland  Jönköping  Kronoberg  Kalmar  Gotland  Blekinge  Skåne  Halland  Västra Götaland  Värmland  Örebro  Västmanland  Dalarna  Gävleborg  Västernorrland  Jämtland  Västerbotten  Norrbotten | 315.1  40.9  44.4  40.5  32.1  21.7  20.8  18.2  52.0  112.7  54.8  66.0  15.5  32.8  49.1  9.8  15.2  11.2  2.6  4.7  2.5 |

**Table S1. Swedish population density estimates 2010, per sq. km. by county.**

Source: Statistics Sweden ([http://www.scb.se](http://www.scb.se/)).
