# Supplementary material for: The Genetic Structure of the Swedish Population
Source: PLoS One. 2011 Aug 4;6(8):e22547. doi: 10.1371/journal.pone.0022547 (PMC3150368; doi:10.1371/journal.pone.0022547)
Supplement: Table S2 — Samples from each study, the number removed and the number remaining. (DOC) [file pone.0022547.s012.doc]

| STUDY | N_Sanples_ | N_Finnish ancestry_ | N_other exclusions_ | N_remaining_ (proportion) |
| --- | --- | --- | --- | --- |
| CAHRES | 764 | 20 | 12 | 732 (0.96) |
| CAPS | 994 | 109 | 35 | 850 (0.86) |
| DGI | 412 | 11 | 3 | 398 (0.97) |
| SCZ-SW | 2494 | 194 | 10 | 2290 (0.92) |
| TWINGENE-SW | 302 | 9 | 3 | 290 (0.96) |
| EIRA | 658 | 31 | 13 | 614 (0.93) |
| **Total** | 5624 | 374 | 76 | 5174 (0.92) |

Table S2. Samples from each study, the number removed and the number remaining
