# Supplementary material for: The Genetic Structure of the Swedish Population
Source: PLoS One. 2011 Aug 4;6(8):e22547. doi: 10.1371/journal.pone.0022547 (PMC3150368; doi:10.1371/journal.pone.0022547)
Supplement: Table S3 — Fsts and λGC 1000s between counties (excluding Gotland county). (DOC) [file pone.0022547.s013.doc]

| Finns | Norrbottens county | Västerbottens county | Jämtlands county | Västernorrlands county | Gävleborgs county | Dalarnas county | Värmlands county | Uppsala county | Västmanlands county | Örebro county | Stockholms county | Södermanlands county | Östergötlands county | Västra Götalands county | Jönköpings county | Kalmar county | Kronobergs county | Hallands county | Blekinge county | Skåne county | HapMap CEU |  |
| --- | --- | --- | --- | --- | --- | --- | --- | --- | --- | --- | --- | --- | --- | --- | --- | --- | --- | --- | --- | --- | --- | --- |
| 0.00532 | 0.002545 | 0.003172 | 0.001552 | 0.001387 | 0.001257 | 0.001638 | 0.00093 | 0.000837 | 0.000745 | 0.000666 | 0.000585 | 0.000697 | 0.000668 | 0.000638 | 0.00075 | 0.000668 | 0.000825 | 0.000717 | 0.000825 | 0.000538 | 0 | HapMap CEU |
| 0.004641 | 0.002183 | 0.002759 | 0.001141 | 0.001003 | 0.000783 | 0.001184 | 0.000552 | 0.000406 | 0.000329 | 0.000289 | 0.000189 | 0.000248 | 0.000182 | 0.000267 | 0.000227 | 0.000231 | 0.000238 | 0.000238 | 0.000227 | 0 | 1.538 | Skåne county |
| 0.004753 | 0.00225 | 0.002793 | 0.001225 | 0.001122 | 0.000887 | 0.00134 | 0.000691 | 0.000474 | 0.00045 | 0.000439 | 0.000288 | 0.000395 | 0.000227 | 0.000401 | 0.00028 | 0.000272 | 0.000237 | 0.000387 | 0 | 1.227 | 1.825 | Blekinge county |
| 0.004762 | 0.002167 | 0.002794 | 0.001227 | 0.001051 | 0.000843 | 0.00129 | 0.000583 | 0.000443 | 0.000407 | 0.000333 | 0.000243 | 0.000324 | 0.00022 | 0.000165 | 0.000139 | 0.000291 | 0.000335 | 0 | 1.387 | 1.238 | 1.717 | Hallands county |
| 0.004515 | 0.002175 | 0.002758 | 0.001176 | 0.000997 | 0.000817 | 0.001257 | 0.000665 | 0.000431 | 0.000389 | 0.000382 | 0.000262 | 0.000284 | 0.0003 | 0.000402 | 0.00025 | 0.000204 | 0 | 1.335 | 1.237 | 1.238 | 1.825 | Kronobergs county |
| 0.004239 | 0.002062 | 0.002655 | 0.001076 | 0.000906 | 0.000695 | 0.001123 | 0.000502 | 0.000337 | 0.000258 | 0.000225 | 0.000115 | 0.000181 | 0.000105 | 0.00027 | 0.000133 | 0 | 1.204 | 1.291 | 1.272 | 1.231 | 1.668 | Kalmar county |
| 0.004457 | 0.002082 | 0.002701 | 0.001127 | 0.00098 | 0.0007 | 0.001185 | 0.000534 | 0.000395 | 0.000336 | 0.000273 | 0.00018 | 0.00021 | 0.000128 | 0.000194 | 0 | 1.133 | 1.25 | 1.139 | 1.28 | 1.227 | 1.75 | Jönköpings county |
| 0.004588 | 0.002106 | 0.002677 | 0.001049 | 0.00094 | 0.000719 | 0.001107 | 0.000434 | 0.000388 | 0.000318 | 0.000234 | 0.000173 | 0.000242 | 0.000185 | 0 | 1.194 | 1.27 | 1.402 | 1.165 | 1.401 | 1.267 | 1.638 | Västra Götalands county |
| 0.004111 | 0.001861 | 0.002446 | 0.000856 | 0.000743 | 0.000521 | 0.00093 | 0.000408 | 0.000207 | 0.000145 | 0.000128 | 0.000062 | 0.0001 | 0 | 1.185 | 1.128 | 1.105 | 1.3 | 1.22 | 1.227 | 1.182 | 1.668 | Östergötlands county |
| 0.003703 | 0.001823 | 0.002429 | 0.000845 | 0.000697 | 0.00046 | 0.000821 | 0.000383 | 0.000159 | 0.000088 | 0.000107 | 0.000046 | 0 | 1.1 | 1.242 | 1.21 | 1.181 | 1.284 | 1.324 | 1.395 | 1.248 | 1.697 | Södermanlands county |
| 0.003808 | 0.001689 | 0.002223 | 0.000734 | 0.000579 | 0.000373 | 0.000765 | 0.000314 | 0.000088 | 0.000064 | 0.000085 | 0 | 1.046 | 1.062 | 1.173 | 1.18 | 1.115 | 1.262 | 1.243 | 1.288 | 1.189 | 1.585 | Stockholms county |
| 0.003796 | 0.001786 | 0.002388 | 0.000897 | 0.000697 | 0.000446 | 0.000786 | 0.000235 | 0.000159 | 0.000085 | 0 | 1.085 | 1.107 | 1.128 | 1.234 | 1.273 | 1.225 | 1.382 | 1.333 | 1.439 | 1.289 | 1.666 | Örebro county |
| 0.003532 | 0.00162 | 0.002175 | 0.000659 | 0.000554 | 0.000293 | 0.000514 | 0.000338 | 0.000093 | 0 | 1.085 | 1.064 | 1.088 | 1.145 | 1.318 | 1.336 | 1.258 | 1.389 | 1.407 | 1.45 | 1.329 | 1.745 | Västmanlands county |
| 0.003501 | 0.001536 | 0.002073 | 0.000727 | 0.000497 | 0.000274 | 0.000699 | 0.000421 | 0 | 1.093 | 1.159 | 1.088 | 1.159 | 1.207 | 1.388 | 1.395 | 1.337 | 1.431 | 1.443 | 1.474 | 1.406 | 1.837 | Uppsala county |
| 0.00404 | 0.001995 | 0.002539 | 0.000952 | 0.000898 | 0.000617 | 0.000876 | 0 | 1.421 | 1.338 | 1.235 | 1.314 | 1.383 | 1.408 | 1.434 | 1.534 | 1.502 | 1.665 | 1.583 | 1.691 | 1.552 | 1.93 | Värmlands county |
| 0.004097 | 0.002139 | 0.002668 | 0.00101 | 0.001096 | 0.000575 | 0 | 1.876 | 1.699 | 1.514 | 1.786 | 1.765 | 1.821 | 1.93 | 2.107 | 2.185 | 2.123 | 2.257 | 2.29 | 2.34 | 2.184 | 2.638 | Dalarnas county |
| 0.003634 | 0.001672 | 0.002209 | 0.000609 | 0.000555 | 0 | 1.575 | 1.617 | 1.274 | 1.293 | 1.446 | 1.373 | 1.46 | 1.521 | 1.719 | 1.7 | 1.695 | 1.817 | 1.843 | 1.887 | 1.783 | 2.257 | Gävleborgs county |
| 0.003854 | 0.001195 | 0.001377 | 0.0006 | 0 | 1.555 | 2.096 | 1.898 | 1.497 | 1.554 | 1.697 | 1.579 | 1.697 | 1.743 | 1.94 | 1.98 | 1.906 | 1.997 | 2.051 | 2.122 | 2.003 | 2.387 | Västernorrlands county |
| 0.004225 | 0.001791 | 0.002147 | 0 | 1.6 | 1.609 | 2.01 | 1.952 | 1.727 | 1.659 | 1.897 | 1.734 | 1.845 | 1.856 | 2.049 | 2.127 | 2.076 | 2.176 | 2.227 | 2.225 | 2.141 | 2.552 | Jämtlands county |
| 0.005341 | 0.001003 | 0 | 3.147 | 2.377 | 3.209 | 3.668 | 3.539 | 3.073 | 3.175 | 3.388 | 3.223 | 3.429 | 3.446 | 3.677 | 3.701 | 3.655 | 3.758 | 3.794 | 3.793 | 3.759 | 4.172 | Västerbottens county |
| 0.00445 | 0 | 2.003 | 2.791 | 2.195 | 2.672 | 3.139 | 2.995 | 2.536 | 2.62 | 2.786 | 2.689 | 2.823 | 2.861 | 3.106 | 3.082 | 3.062 | 3.175 | 3.167 | 3.25 | 3.183 | 3.545 | Norrbottens county |
| 0 | 5.45 | 6.341 | 5.225 | 4.854 | 4.634 | 5.097 | 5.04 | 4.501 | 4.532 | 4.796 | 4.808 | 4.703 | 5.111 | 5.588 | 5.457 | 5.239 | 5.515 | 5.762 | 5.753 | 5.641 | 6.32 | Finns |

Table S3. F_st_s and λ_GC 1000_s between counties (excluding Gotland county)
