# Supplementary material for: The Genetic Structure of the Swedish Population
Source: PLoS One. 2011 Aug 4;6(8):e22547. doi: 10.1371/journal.pone.0022547 (PMC3150368; doi:10.1371/journal.pone.0022547)
Supplement: Table S4 — Fsts and λGC 1000s between national areas, using data from the SCZ-SW study only. Differences above 0.008 (Fst) or 1.8 (λGC 1000) have been made bold. (DOC) [file pone.0022547.s014.doc]

|  | HapMap CEU | Southern Sweden | Småland with the islands | Western Sweden | Stockholm | East Middle Sweden | North Middle Sweden | Middle Norrland | Upper Norrland | Finns |
| --- | --- | --- | --- | --- | --- | --- | --- | --- | --- | --- |
| HapMap CEU |  | 0.000531 | 0.000673 | 0.000764 | 0.000678 | 0.000714 | **0.001036** | **0.001274** | **0.002024** | **0.005320** |
| Southern Sweden | 1.531000 |  | 0.000144 | 0.000227 | 0.000204 | 0.000266 | 0.000594 | **0.000872** | **0.001656** | **0.004644** |
| Småland with the islands | 1.673000 | 1.144000 |  | 0.000119 | 0.000116 | 0.000188 | 0.000487 | **0.000804** | **0.001536** | **0.004308** |
| Western Sweden | 1.764000 | 1.227000 | 1.119000 |  | 0.000260 | 0.000277 | 0.000591 | **0.000929** | **0.001625** | **0.004611** |
| Stockholm | 1.678000 | 1.204000 | 1.116000 | 1.260000 |  | 0.000016 | 0.000216 | 0.000431 | **0.001164** | **0.003696** |
| East Middle Sweden | 1.714000 | 1.266000 | 1.188000 | 1.277000 | 1.016000 |  | 0.000205 | 0.000512 | **0.001179** | **0.003620** |
| North Middle Sweden | **2.036000** | 1.594000 | 1.487000 | 1.591000 | 1.216000 | 1.205000 |  | 0.000424 | **0.001148** | **0.003683** |
| Middle Norrland | **2.274000** | **1.872000** | **1.804000** | **1.929000** | 1.431000 | 1.512000 | 1.424000 |  | 0.000751 | **0.003903** |
| Upper Norrland | **3.024000** | **2.656000** | **2.536000** | **2.625000** | **2.164000** | **2.179000** | **2.148000** | 1.751000 |  | **0.003891** |
| Finns | **6.320000** | **5.644000** | **5.308000** | **5.611000** | **4.696000** | **4.620000** | **4.683000** | **4.903000** | **4.891000** |  |

Table S4. F_st_s and λ_GC 1000_s between national areas, using data from the SCZ-SW study only. Differences above 0.008 (F_st_) or 1.8 (λ_GC 1000_) have been made bold.
