# Supplementary material for: The Genetic Structure of the Swedish Population
Source: PLoS One. 2011 Aug 4;6(8):e22547. doi: 10.1371/journal.pone.0022547 (PMC3150368; doi:10.1371/journal.pone.0022547)
Supplement: Table S5 — Between-study Fst comparison. Stockholm county above and Skåne county below. (DOC) [file pone.0022547.s015.doc]

| *Stockholm county* | SCZ-SW | CAHRES | CAPS | TWINGENE-SW | EIRA |
| --- | --- | --- | --- | --- | --- |
| SCZ-SW |  | 0.000012 | 0.000125 | 0.000205 | 0.000073 |
| CAHRES |  |  | 0.000074 | 0.000102 | -0.000007 |
| CAPS |  |  |  | 0.000131 | 0.000134 |
| TWINGENE-SW |  |  |  |  | -0.000039 |
| EIRA |  |  |  |  |  |
|  |  |  |  |  |  |
| *Skåne county* | SCZ-SW | CAHRES | TWINGENE-SW | DGI | EIRA |
| SCZ-SW |  | 0.000062 | 0.000086 | 0.000045 | 0.000145 |
| CAHRES |  |  | 0.000097 | 0.000205 | 0.000152 |
| TWINGENE-SW |  |  |  | 0.000169 | 0.000079 |
| DGI |  |  |  |  | 0.00026 |
| EIRA |  |  |  |  |  |

Table S5. Between-study F_st_ comparison. Stockholm county above and Skåne county below.
